# Supplementary material for: Chromosome drives via CRISPR-Cas9 in yeast
Source: Nat Commun. 2020 Aug 28;11:4344. doi: 10.1038/s41467-020-18222-0 (PMC7455567; doi:10.1038/s41467-020-18222-0)
Supplement: Supplementary file 1 — Supplementary Information [file 41467_2020_18222_MOESM1_ESM.pdf]

Supplementary information

Chromosome drives via CRISPR-Cas9 in yeast

Xu et al.

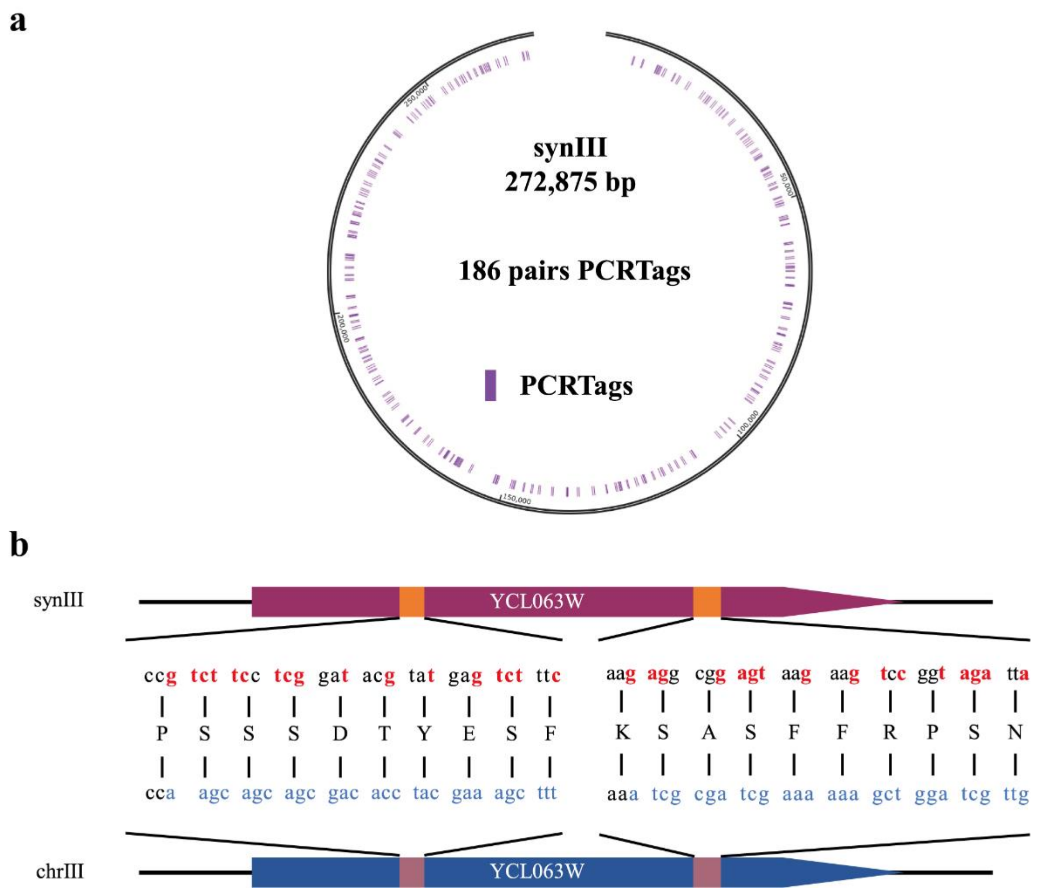

Supplementary Figure 1. The design of PCRTags in YCL063W. a, Summary of synthetic PCRTags of synIII. The purple frames represent the synthetic PCRTags of synIII. b, The synonymous mutation of YCL063W does not change the amino acid sequence, but changes the DNA sequence. The blue bases represent the primer sequences of wild-type PCRTags, and the red bases represent the base mutations on the synthetic PCRTags of synIII.

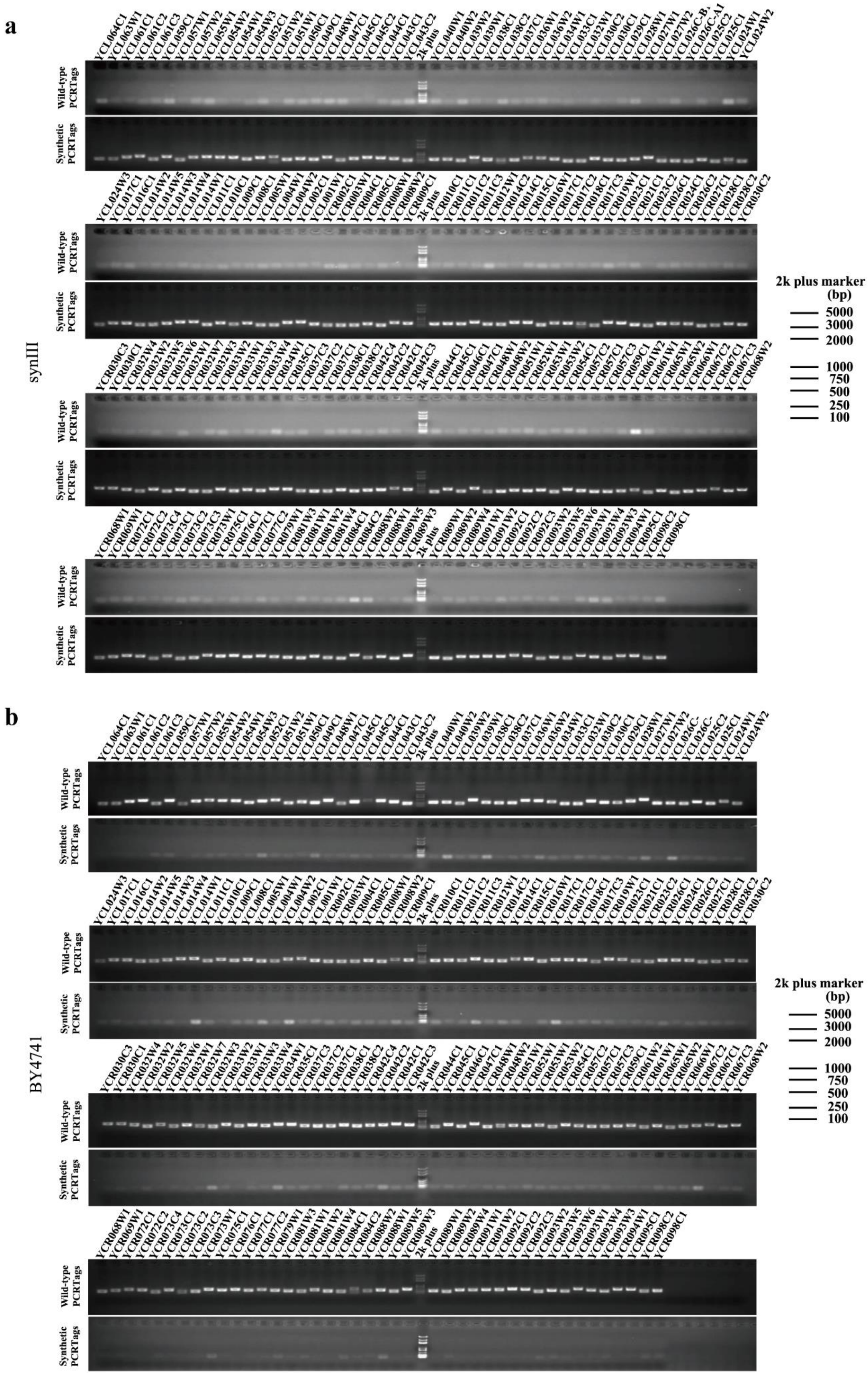

(Next page for figure legend)

Supplementary Figure 2. The PCRTag analysis of diploid controls. a, All synthetic and native PCRtags were tested in diploid control (synIII). b, All synthetic and native PCRtags were tested in diploid control (BY4741). Source data are provided as a Source Data file.

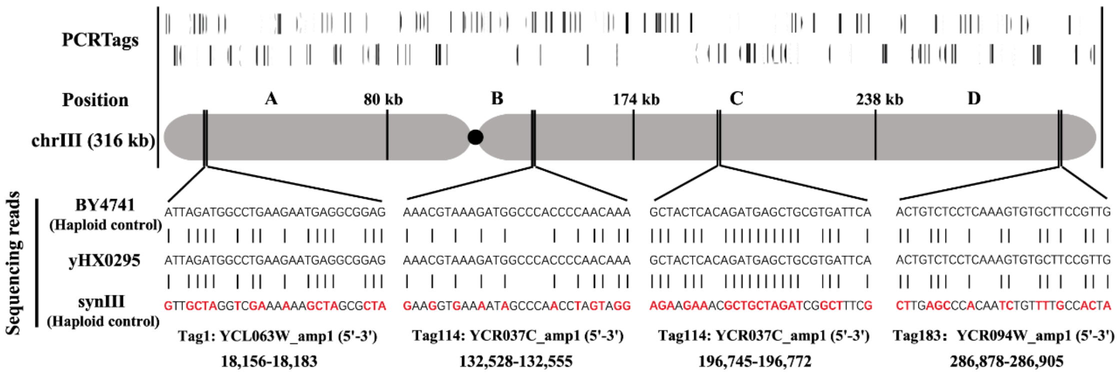

Supplementary Figure 3. Whole-genome sequencing analysis of synIII elimination in yHX0295. The base alignment of the BY4741, yHX0295 and synIII strains is shown. The red bases represent the mismatched bases of PCRTags in the haploid control (synIII). All the PCRTags in yHX0186 matched with the haploid control (BY4741), and four of them were are displayed.

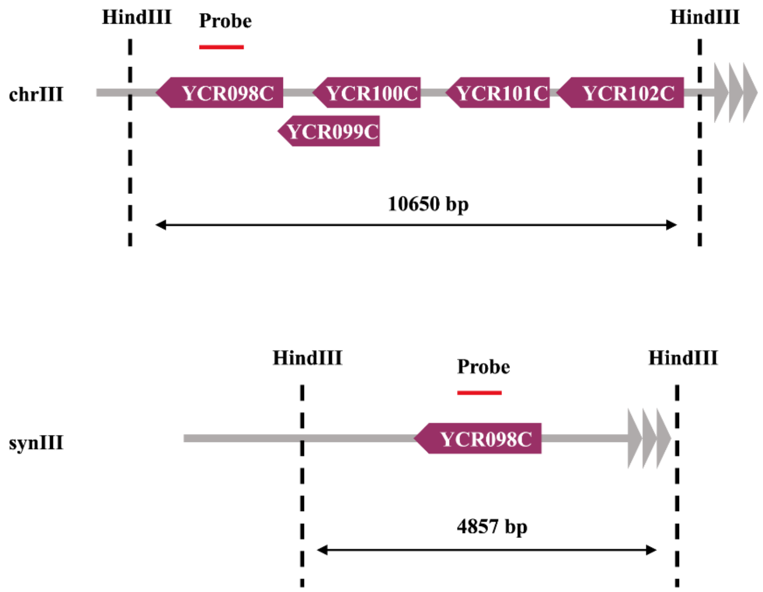

Supplementary Figure 4. The scheme of southern blot analysis of synIII elimination. In chrIII, with the digestion of HindIII, the 10650 bp fragment with YCR098C was yielded. And in synIII, the 4857 bp right arm with YCR098C was cut. The southern bolt was used to distinguish two fragments from chrIII and synIII.

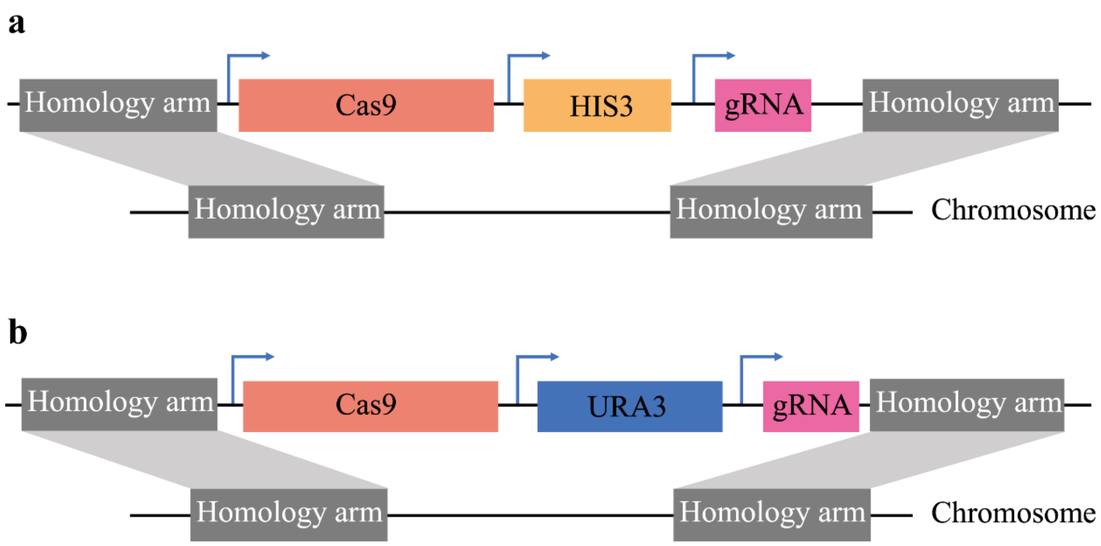

Supplementary Figure 5. Transgene maps for the Cas9 and gRNAs throughout the work. a, The transgene map for the integration of CRISPR cassette onto synX and chrX. The HIS3 nutrition label is inserted between Cas9 and gRNA, and this cassette is integrated into the chromosome. For synX and chrX, the CRISPR cassette is integrated between YJR094C and YJR094W-A. b, The transgene map for the integration of CRISPR cassette onto synXII and Y12 's chrXIV. Since synXII strain and Y12 strain only have URA3 nutrition label, URA3 nutrition label was inserted between Cas9 and gRNA to facilitate label recovery. For synXII, the CRISPR cassette was integrated between YLR122C and YLR125W. For chrXIV, the CRISPR cassette was integrated between YNL161W and YNL160W.

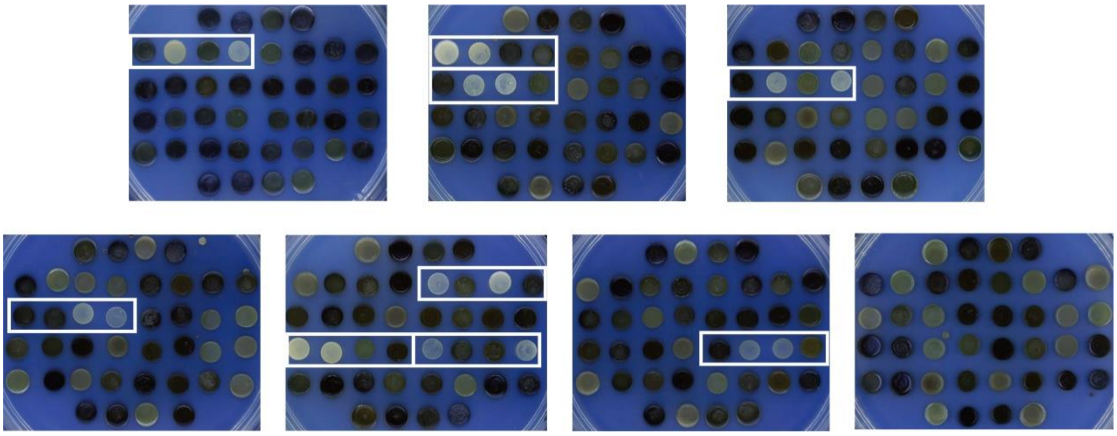

Supplementary Figure 6. The drive efficiency analysis by the color of spores. 70 diploid strains were randomly selected to sporulate and disintegrated them into tetrads. Spores above were from 70 tetrads. Among 70 tetrads, 61 of them yielded a 0:4 white/purple ratio spores and 9 of them yielded a 2:2 white/purple ratio spores, indicating the efficiency of chromosome drives turned to be 87.14%. Source data are provided as a Source Data file.

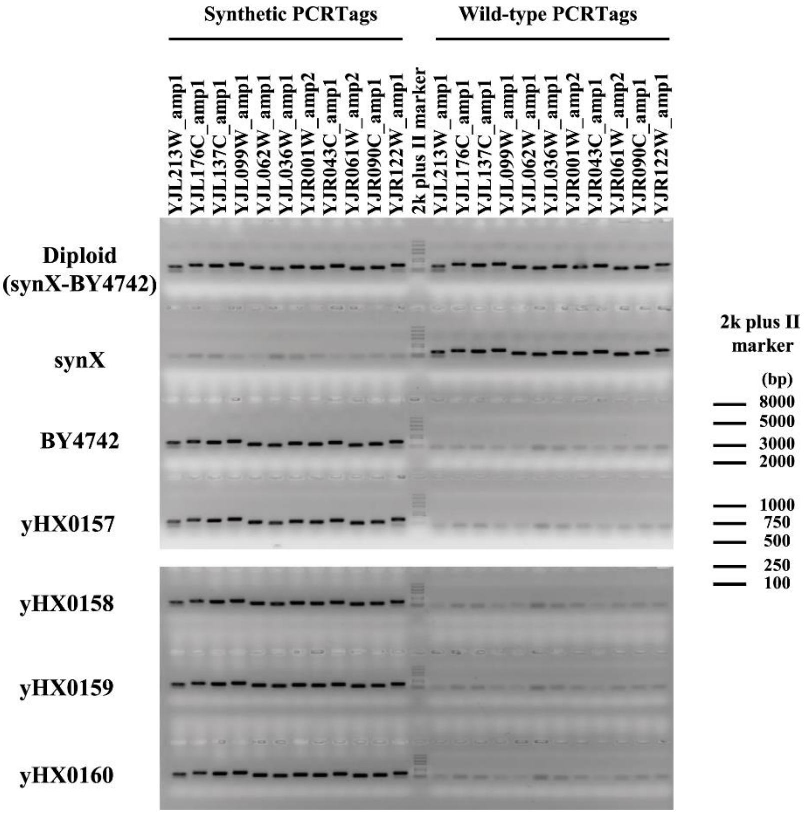

Supplementary Figure 7. PCRTag analysis of spores from synX elimination. SynX strain, BY4742 strain and the synX&BY4742 diploid strain were used as negative and positive control. 12 synthetic PCRtags and 12 wild-type PCRtags (YJL213W_amp1, YJL176C_amp1, YJL137C_amp1, YJL099W_amp1, YJL062W_amp1, YJL036W_amp1, YJR001W_amp2, YJR043C_amp1, YJR061W_amp2, YJR090C_amp1, YJR122W_amp1, YJR143C_amp1) were used in this trail. Source data are provided as a Source Data file.

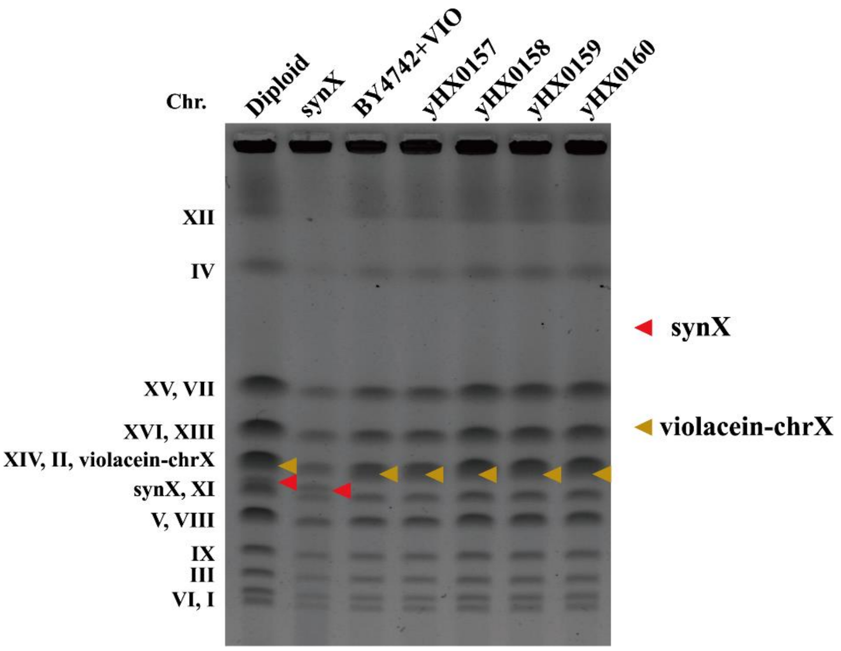

Supplementary Figure 8. PFGE analysis of spores from synX elimination. SynX strain, BY4742 strain with violacein pathway and the synX&BY4742 diploid strain with violacein pathway were used as control. The length of violacein-chrX is longer than synX. And the longer chromosome, violacein-chrX, was contained in all four spores. PFGE analysis revealed synX was lost and chrX with violacein pathway existed in all four spores. Source data are provided as a Source Data file.

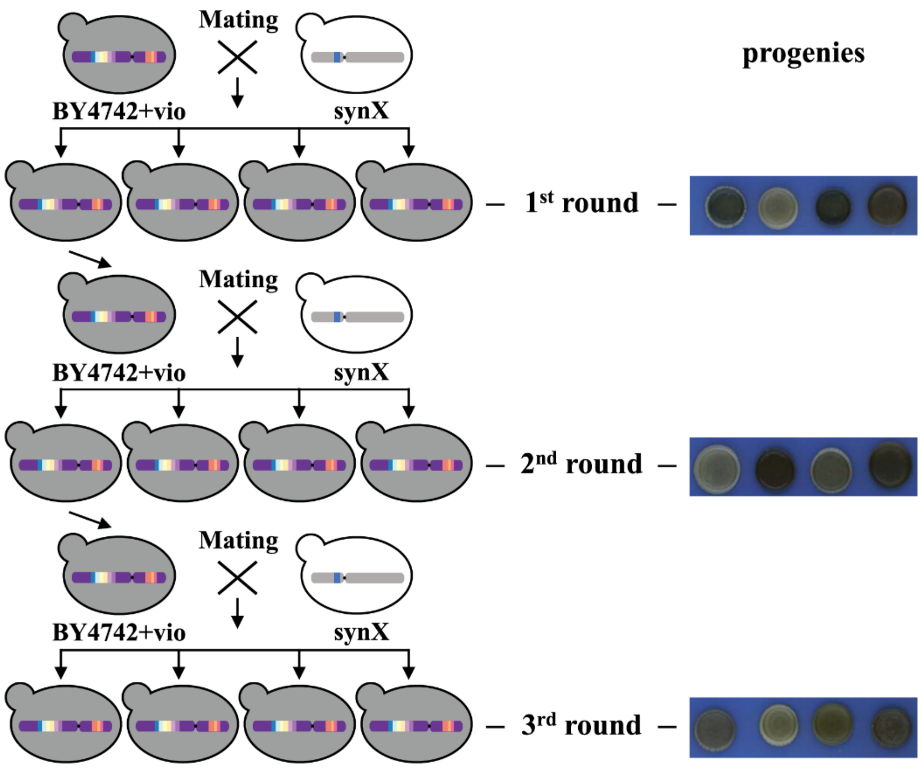

Supplementary Figure 9. Workflow for multiple rounds of chromosome dives in yeast. Each round of drive was performed by mating BY4742 from the last round of drive and synX strain. Spores on the right were dissected from one driven diploid strain for each round. Source data are provided as a Source Data file.

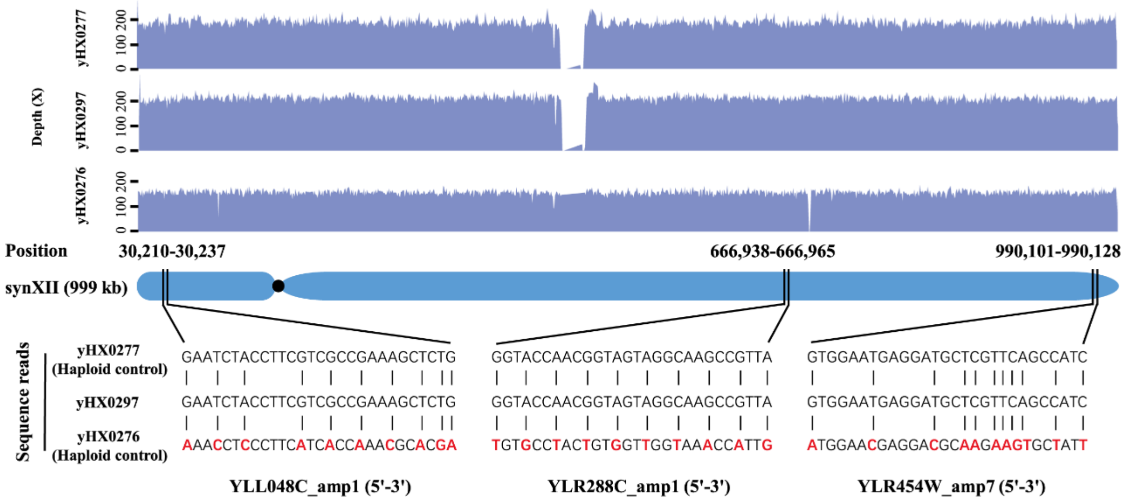

Supplementary Figure 10. Whole-genome sequencing analysis of chrXII elimination in yHX0297. The coverage map showed the coverage depth of whole-genome sequencing. The bases alignment of yHX0277, yHX0297 and yHX0276 was showed below the coverage map. The mismatch bases in yHX0276 compared with yHX0297 were highlighted in red. All synthetic PCRTags in yHX0297 matched with that in yHX0277, and three of them were displayed in the figure.

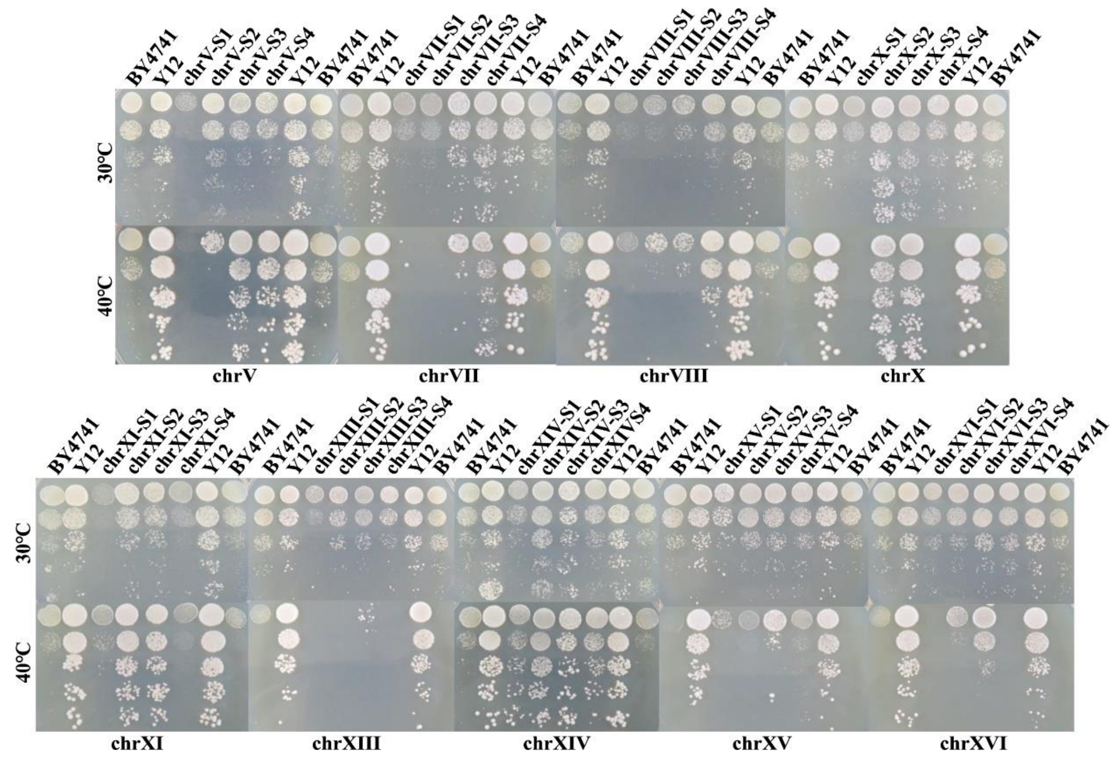

Supplementary Figure 11. Serial dilution assays analysis of the thermotolerance-related chromosome of Y12. 9 chromosomes were selected to test thermotolerant trait at 30 ℃ and 40 ℃. Chromosome of BY4741 was eliminated in the diploid obtained by mating BY4741 with Y12, and four spores from one diploid strain were used to test phenotype. The result showed that all spores containing chr XIV of Y12 were more thermotolerant compared to controls, indicating chrXIV of Y12 is a thermotolerance-related chromosome. Source data are provided as a Source Data file.

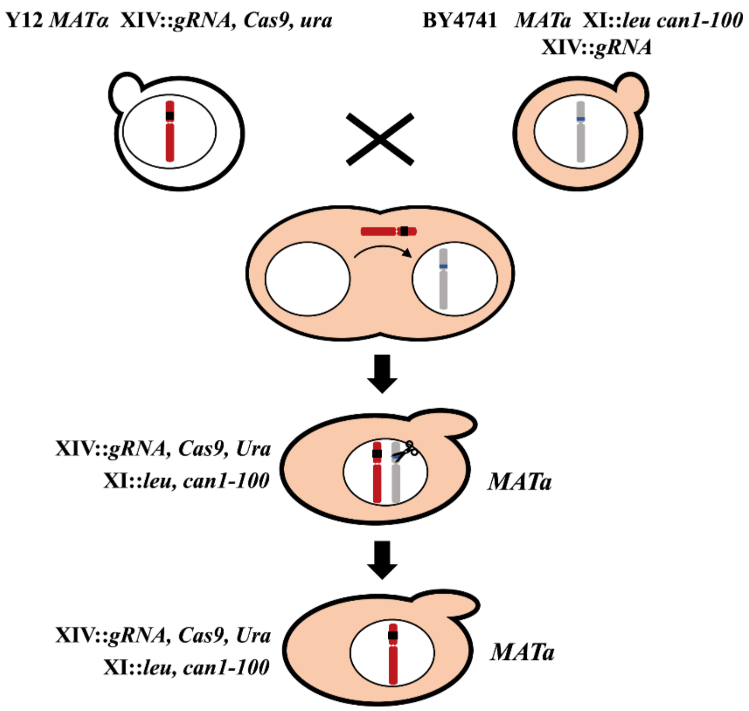

Supplementary Figure 12. Workflow for constructing heterozygous haploid. CRISPR/Cas9 system was inserted into chrXIV with URA3 marker in Y12. In BY4741, synthetic CRISPR targeted sequence (5’-GTTGCAAATGCTCCGTCGACGGG-3’) was inserted into chrXIV, and *LEU2* was integrated into chrXI, besides, *karI* was mutated and *can-100* was deleted. Mating two strains described above, chrXIV of Y12 was transferred into BY4741, and corresponding chrXIV of BY4741 will be eliminated by CRISPR/Cas9. The transferred strains can be selected by SC-LEU-URA+canavanine selective media.

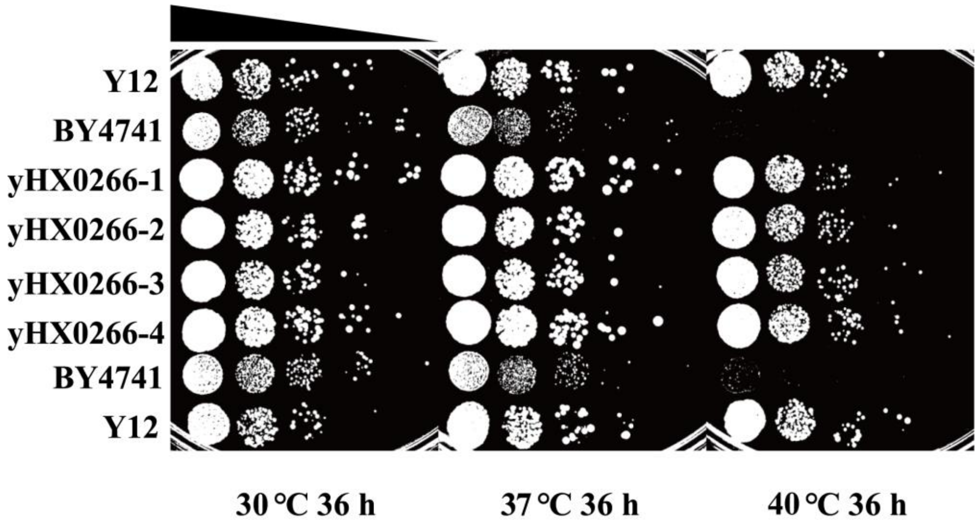

Supplementary Figure 13. Confirmation of thermotolerance -related chromosome in Y12. Four strains of heterozygous haploid strain yHX0266 were selected for the test with Y12 and BY4741 at 30 °C, 37 °C and 40 °C. Serial dilution assay analysis showed yHX0266 strains grew better than BY4741 at 40 °C. Source data are provided as a Source Data file.

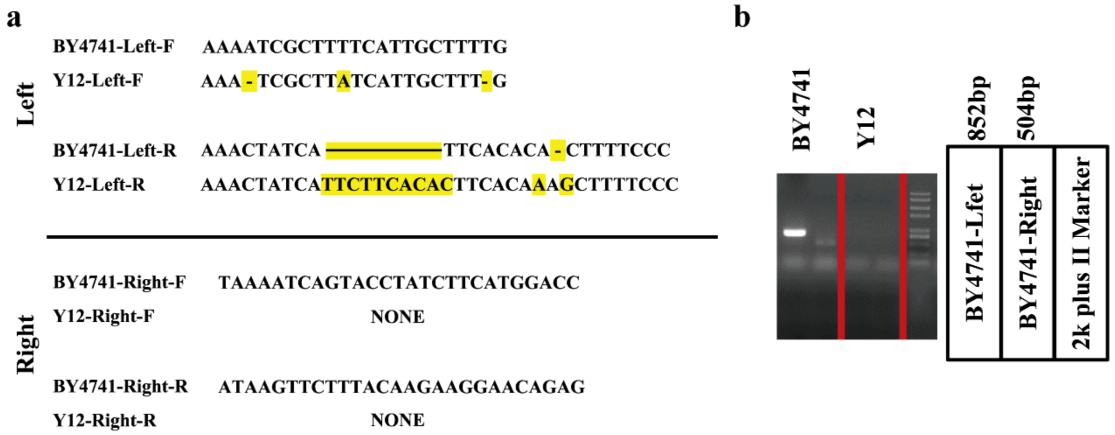

Supplementary Figure 14. PCRTags for assessing elimination of chrXIV of BY4741 in a diploid strain obtained by mating BY4742 with Y12. a, Two specific DNA sequence regions were selected from both end of chrXIV. The primers were designed in two regions which were matched in BY4741 and mismatched in Y12. Yellow frames represents the mismatched bases in Y12. b, PCR analysis by two pairs of primers, the result showed two specific regions can be amplified in BY4741 and displayed negative in Y12, indicating that these two pairs of primers were suitable PCRTags to distinguish between BY4741 and Y12. Source data are provided as a Source Data file.

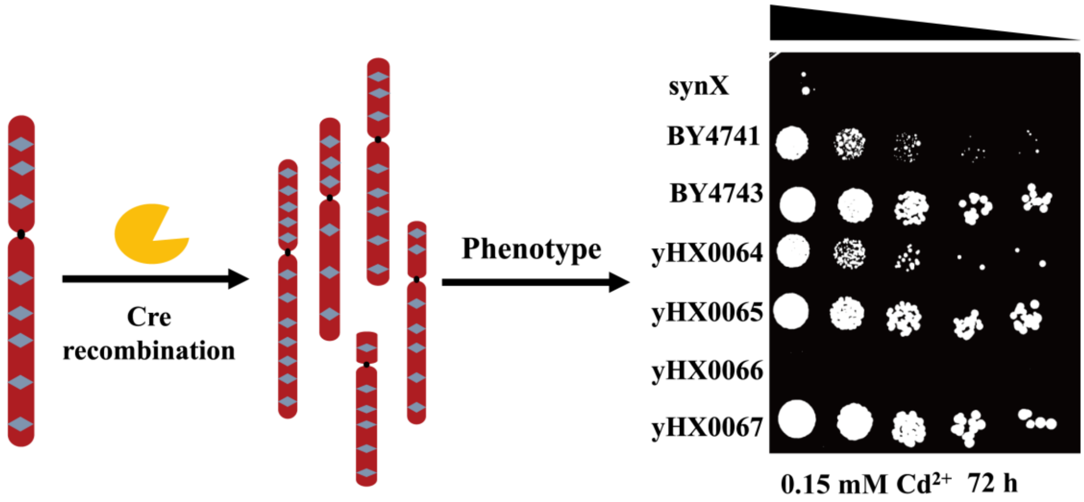

Supplementary Figure 15. The serial dilution assay analysis of chromium resistance of SCRaMbLEd strains. The SCRaMbLEd strains (yHX0064, yHX0065, yHX0066, yHX0067) displayed a visible improvement of chromium resistance than synX strain. Source data are provided as a Source Data file.

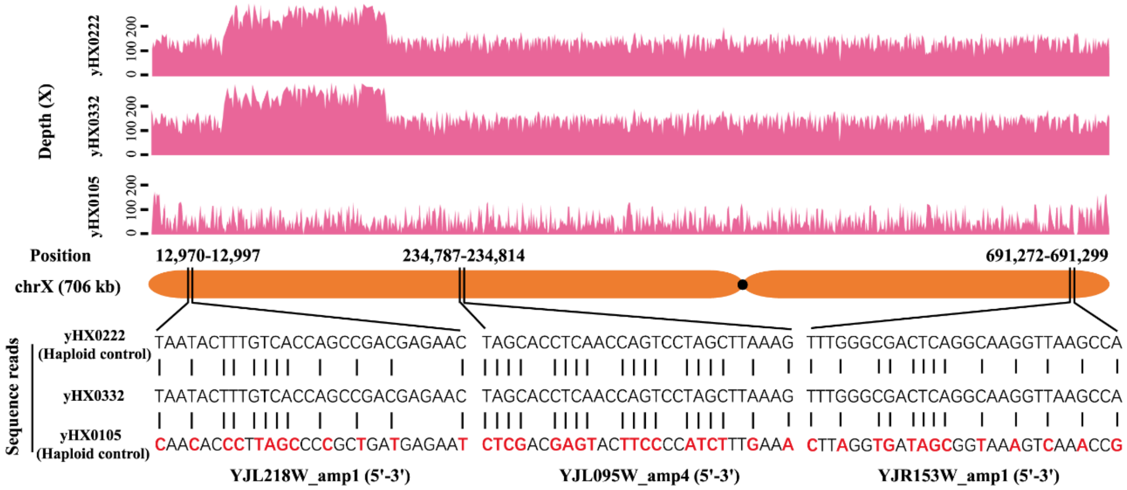

Supplementary Figure 16. Whole-genome sequencing analysis of chrX elimination in yHX0332. The coverage map showed the coverage depth of whole-genome sequencing. The bases alignment of yHX0222, yHX0332 and yHX0105 was showed below the coverage map. The mismatch bases of yHX0105 compared with yHX0332 were highlighted in red. All synthetic PCRTags in yHX0332 matched with that in yHX0222, and three of them were displayed in the figure.

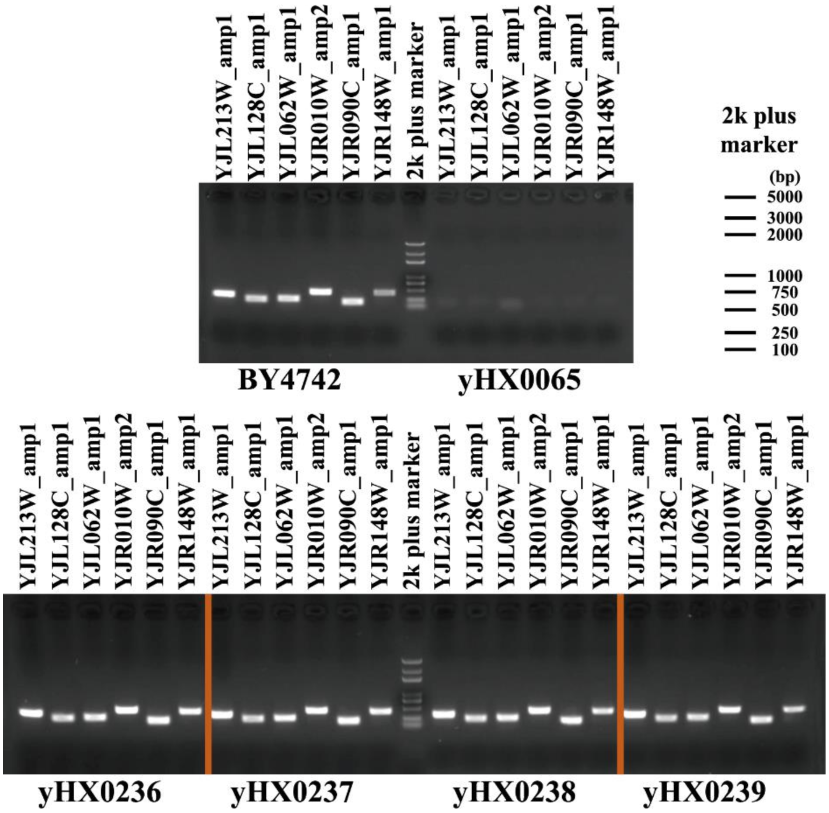

Supplementary Figure 17. PCRTag analysis of spores within SCRaMbLEd synX from a driven strain. Six synthetic PCRTags (YJL213W_amp1, YJL128C_amp1, YJL062W_amp1, YJR010W_amp2, YJR090C_amp1, YJR148W_amp1) across the synX were used for verification. Each lane represents one strain, BY4742 and yHX0065 were used as the control. The result showed that all spores (yHX0236, yHX0237, yHX238 and yHX0239) had synthetic PCRTags, indicating that SCRaMbLEd synX reminded in spores. Source data are provided as a Source Data file.

Cut site	Mating non-available colony number	Mating available colony number	Total colony number	efficiency of synIII elimination
LoxPsym site	59	219	278	78.78%

Supplementary Table 1. The number of colonies in mating type test experiment. Total colonies and mating available were counted at corresponding plates.

Parallel	cutting position						
	50bp	100bp	150bp	200bp	1k	L-M	L-T
1	92.5% (111/120)	90.83% (109/120)	78.33% (94/120)	84.17% (101/120)	0.00% (0/120)	0.00% (0/120)	0.00% (0/120)
2	85.00% (102/120)	80.83% (97120)	83.33% (100/120)	75.00% (90/120)	0.00% (0/120)	0.00% (0/120)	0.00% (0/120)
3	90.00% (108/120)	85.83% (103/120)	85.00% (102/120)	81.67% (98/1200	0.00% (0/120)	0.00% (0/120)	0.00% (0/120)

Supplementary Table 2. The elimination efficiency of strains whose synX was eliminated at each parallel. 120 strains of each replicates were selected for each cutting position.

chromosome	chromosome length	targeted sequence	number of tested strains	deletion efficiency
synX	707kb	5’-GTTGCAAATG CTCCGTCGAC GGG-3’	61	87.14% (61/70)
synXII	976kb (without rDNA)		20	20.83% (20/96)
chrXIV	784kb		39	40.63% (39/96)
chrX	745kb		84	87.5% (84/96)

Supplementary Table 3. Deletion efficiency for different chromosomes. All the chromosomes were driven by cutting at synthetic CRISPR targeted sequence (5’-GTTGCAAATGCTCCGTCGAC GGG-3’) 50bp away from centromere on the left arm of each chromosome. For synX, the efficiency was tested by counting the purple color spores, and synXII, chrXIV and chrX were analyzed by PCRTags.

range (kb)	SNPs				
	Y12	yHX0285	yHX0286	yHX0287	yHX0288
26	48	43	44	48	44
52	51	51	50	51	51
78	139	133	139	135	136
104	67	67	67	67	67
130	180	173	170	176	174
156	162	156	156	157	159
182	128	128	128	128	128
208	137	134	135	135	136
234	97	96	96	97	97
260	97	97	95	96	98
286	119	117	119	118	119
312	172	163	162	167	170
338	125	125	125	125	125
364	199	197	192	197	199
390	123	123	119	123	123
416	66	65	65	65	64
442	167	163	164	166	166
468	116	116	116	115	116
494	142	140	139	141	140
520	140	133	133	135	136
546	109	109	109	108	110
572	52	48	49	54	53
598	58	57	57	57	57
624	96	94	94	94	94
650	91	91	90	91	91
676	173	166	165	172	171
702	131	130	129	131	131
728	181	170	181	183	185
754	213	208	209	211	213
780	141	133	140	145	140

Supplementary Table 4. The number of SNPs that was compared with chrXIV of BY4741. SNPs were counted every 26kb.

eliminated chromosome	strain	undriven types of target region			description
		Target unedited	HR	NHEJ	
synX	yHX0322		√		HDR happened across the centromere, the range is about 359kb
	yHX0323		√		HDR happened across the centromere, the range is about 11kb
	yHX0324		√		HDR happened across the centromere, the range is about 23kb
	yHX0325		√		HDR happened across the centromere, the range is about 422kb
	yHX0326		√		HDR happened across the centromere, the range is about 5kb
	yHX0327		√		HDR happened across the centromere, the range is about 3kb
	yHX0328		√		HDR happened across the centromere, the range is about 39kb
	yHX0329		√		HDR happened across the centromere, the range is about 20kb
	yHX0330		√		HDR happened across the centromere, the range is about 39kb
	yHX0331		√		HDR happened across the centromere, the range is about 15kb
chrXII	yHX0302	√			The synthetic CRISPR target, chrXII and synXII maintain intact
	yHX0303	√			The synthetic CRISPR target, chrXII and synXII maintain intact
	yHX0304	√			The synthetic CRISPR target, chrXII and synXII maintain intact

	yHX0305	√			The synthetic CRISPR target, chrXII and synXII maintain intact
	yHX0306	√			The synthetic CRISPR target, chrXII and synXII maintain intact
	yHX0307	√			The synthetic CRISPR target, chrXII and synXII maintain intact
	yHX0308	√			The synthetic CRISPR target, chrXII and synXII maintain intact
	yHX0309	√			The synthetic CRISPR target, chrXII and synXII maintain intact
	yHX0310	√			The synthetic CRISPR target, chrXII and synXII maintain intact
	yHX0311		√		HDR happened across the centromere, the range is about 908kb
chrXIV of BY4742	yHX0312		√		The synthetic CRISPR target was lost by the nearby HDR
	yHX0313		√		The synthetic CRISPR target was lost by the nearby HDR
	yHX0314		√		HDR happened across the centromere, the range is about 5kb
	yHX0315		√		HDR happened across the centromere, the range is about 53kb
	yHX0316		√		HDR happened across the centromere, the range is about 5kb
	yHX0317		√		The left arm of chrXIV of BY4742 was lost with the duplication of the left arm of chrXIV of Y12
	yHX0318		√		The right arm of chrXIV of BY4742 was lost with the duplication of the right arm of chrXIV of Y12

	yHX0319		√		The left arm of chrXIV of BY4742 was lost with the duplication of the left arm of chrXIV of Y12
	yHX0320		√		The left arm of chrXIV of BY4742 was lost with the duplication of the left arm of chrXIV of Y12
	yHX0321		√		HDR happened across the centromere, the range is about 4kb
chrX	yHX0336		√		HDR happened across the centromere, the range is about 36kb
	yHX0337		√		HDR happened across the centromere, the range is about 8kb
	yHX0338		√		HDR happened across the centromere, the range is about 70kb
	yHX0339		√		HDR happened across the centromere, the range is about 85kb
	yHX0340		√		HDR happened across the centromere, the range is about 18kb
	yHX0341		√		HDR happened across the centromere, the range is about 14kb
	yHX0342		√		HDR happened across the centromere, the range is about 35kb
	yHX0343		√		HDR happened across the centromere, the range is about 5kb
	yHX0344		√		HDR happened across the centromere, the range is about 16kb
	yHX0345		√		HDR happened across the centromere, the range is about 4kb

(Next page for figure legend)

Supplementary Table 5. The undriven types of target region in escaped strains for the elimination of synX, chrXII, chrXIV of BY4742 and chrX.
