# Supplementary material for: Chromosome drives via CRISPR-Cas9 in yeast
Source: Nat Commun. 2020 Aug 28;11:4344. doi: 10.1038/s41467-020-18222-0 (PMC7455567; doi:10.1038/s41467-020-18222-0)
Supplement: Supplementary file 3 — Description of Additional Supplementary Files [file 41467_2020_18222_MOESM3_ESM.pdf]

Title: Supplementary Data 1.

Description: Yeast strains used in this study.

Title: Supplementary Data 2.

Description: Plasmids used in this study.

Title: Supplementary Data 3.

Description: Primers used in this study.
