# Supplementary material for: Chromosome drives via CRISPR-Cas9 in yeast
Source: Nat Commun. 2020 Aug 28;11:4344. doi: 10.1038/s41467-020-18222-0 (PMC7455567; doi:10.1038/s41467-020-18222-0)
Supplement: Supplementary file 5 — Supplementary Data 2 [file 41467_2020_18222_MOESM5_ESM.docx]

| pRS42H with one gRNA site | 5’-TCTTTGAAAAGATAATGTATGATTATGCTTTCACTCATATTTA  TACAGAAACTTGATGTTTTCTTTCGAGTATATACAAGGTGATTACATGTACGTTTGAAGTACAACTCTAGATTTTGTAGTGCCCTCTTGGGCTAGCGGTAAAGGTGCGCATTTTTTCACACCCTACAATGTTCTGTTCAAAAGATTTTGGTCAAACGCTGTAGAAGTGAAAGTTGGTGCGCATGTTTCGGCGTTCGAAACTTCTCCGCAGTGAAAGATAAATGATC-[Insert 20 nucleotide gRNA protospacer]-GTTTTAGAGCTAGAAATAGCAAGTTAAAATAAGGCTAGTCCGTTATCAACTTGAAAAAGTGGCACCGAGTCGGTGGTGCTTTTTTTGTTTTTTATGTCTGGTACCaattcgccctatagtgagtcgtattacgcgcgctcactggccgtcgttttacaacgtcgtgactgggaaaaccctggcgttacccaacttaatcgccttgcagcacatccccctttcgccagctggcgtaatagcgaagaggcccgcaccgatcgcccttcccaacagttgcgcagcctgaatggcgaatggcgcgacgcgccctgtagcggcgcattaagcgcggcgggtgtggtggttacgcgcagcgtgaccgctacacttgccagcgccctagcgcccgctcctttcgctttcttcccttcctttctcgccacgttcgccggctttccccgtcaagctctaaatcgggggctccctttagggttccgatttagtgctttacggcacctcgaccccaaaaaacttgattagggtgatggttcacgtagtgggccatcgccctgatagacggtttttcgccctttgacgttggagtccacgttctttaatagtggactcttgttccaaactggaacaacactcaaccctatctcggtctattcttttgatttataagggattttgccgatttcggcctattggttaaaaaatgagctgatttaacaaaaatttaacgcgaattttaacaaaatattaacgtttacaatttcctgatgcggtattttctccttacgcatctgtgcggtatttcacaccgccgtcccaaaaccttctcaagcaaggttttcagtataatgttacatgcgtacacgcgtctgtacagaaaaaaaagaaaaatttgaaatataaataacgttcttaatactaacataactataaaaaaataaatagggacctagacttcaggttgtctaactccttccttttcggttagagcggatgtggggggagggcgtgaatgtaagcgtgacataactaattacatgactcgagattattcctttgccctcggacgagtgctggggcgtcggtttccactatcggcgagtacttctacacagccatcggtccagacggccgcgcttctgcgggcgatttgtgtacgcccgacagtcccggctccggatcggacgattgcgtcgcatcgaccctgcgcccaagctgcatcatcgaaattgccgtcaaccaagctctgatagagttggtcaagaccaatgcggagcatatacgcccggagccgcggcgatcctgcaagctccggatgcctccgctcgaagtagcgcgtctgctgctccatacaagccaaccacggcctccagaagaagatgttggcgacctcgtattgggaatccccgaacatcgcctcgctccagtcaatgaccgctgttatgcggccattgtccgtcaggacattgttggagccgaaatccgcgtgcacgaggtgccggacttcggggcagtcctcggcccaaagcatcagctcatcgagagcctgcgcgacggacgcactgacggtgtcgtccatcacagtttgccagtgatacacatggggatcagcaatcgcgcatatgaaatcacgccatgtagtgtattgaccgattccttgcggtccgaatgggccgaacccgctcgtctggctaagatcggccgcagcgatcgcatccatggcctccgcgaccggctgcagaacagcgggcagttcggtttcaggcaggtcttgcaacgtgacaccctgtgcacggcgggagatgcaataggtcaggctctcgctgaattccccaatgtcaagcacttccggaatcgggagcgcggccgatgcaaagtgccgataaacataacgatctttgtagaaaccatcggcgcagctatttacccgcaggacatatccacgccctcctacatcgaagctgaaagcacgagattcttcgccctccgagagctgcatcaggtcggagacgctgtcgaacttttcgatcagaaacttctcgacagacgtcgcggtgagttcaggctttttacccatggttgtttatgttcggatgtgatgtgagaactgtatcctagcaagattttaaaaggaagtatatgaaagaagaacctcagtggcaaatcctaaccttttatatttctctacaggggcgcggcgtggggacaattcaacgcgtctgtgaggggagcgtttccctgctcgcaggtccgcagcgaggagccgtaatttttgcttcgcgccgtgcggccatcaaaatgtatggatgcaaatgattatacatggggatgtatgggctaaatgtacgggcgacagtcacatcatgcccctgagctgcgcacgtcaagactgtcaaggagggtattctgggcctccatgtcgctgcactctcagtacaatctgctctgatgccgcatagttaagccagccccgacacccgccaacacccgctgacgcgccctgacgggcttgtctgctcccggcatccgcttacagacaagctgtgaccgtctccgggagctgcatgtgtcagaggttttcaccgtcatcaccgaaacgcgcgagacgaaagggcctcgtgatacgcctatttttataggttaatgtcatgataataatggtttcttagtatgatccaatatcaaaggaaatgatagcattgaaggatgagactaatccaattgaggagtggcagcatatagaacagctaaagggtagtgctgaaggaagcatacgataccccgcatggaatgggataatatcacaggaggtactagactacctttcatcctacataaatagacgcatataagtacgcatttaagcataaacacgcactatgccgttcttctcatgtatatatatatacaggcaacacgcagatataggtgcgacgtgaacagtgagctgtatgtgcgcagctcgcgttgcattttcggaagcgctcgttttcggaaacgctttgaagttcctattccgaagttcctattctctagaaagtataggaacttcagagcgcttttgaaaaccaaaagcgctctgaagacgcactttcaaaaaaccaaaaacgcaccggactgtaacgagctactaaaatattgcgaataccgcttccacaaacattgctcaaaagtatctctttgctatatatctctgtgctatatccctatataacctacccatccacctttcgctccttgaacttgcatctaaactcgacctctacattttttatgtttatctctagtattactctttagacaaaaaaattgtagtaagaactattcatagagtgaatcgaaaacaatacgaaaatgtaaacatttcctatacgtagtatatagagacaaaatagaagaaaccgttcataattttctgaccaatgaagaatcatcaacgctatcactttctgttcacaaagtatgcgcaatccacatcggtatagaatataatcggggatgcctttatcttgaaaaaatgcacccgcagcttcgctagtaatcagtaaacgcgggaagtggagtcaggctttttttatggaagagaaaatagacaccaaagtagccttcttctaaccttaacggacctacagtgcaaaaagttatcaagagactgcattatagagcgcacaaaggagaaaaaaagtaatctaagatgctttgttagaaaaatagcgctctcgggatgcatttttgtagaacaaaaaagaagtatagattctttgttggtaaaatagcgctctcgcgttgcatttctgttctgtaaaaatgcagctcagattctttgtttgaaaaattagcgctctcgcgttgcatttttgttttacaaaaatgaagcacagattcttcgttggtaaaatagcgctttcgcgttgcatttctgttctgtaaaaatgcagctcagattctttgtttgaaaaattagcgctctcgcgttgcatttttgttctacaaaatgaagcacagatgcttcgttcaggtggcacttttcggggaaatgtgcgcggaacccctatttgtttatttttctaaatacattcaaatatgtatccgctcatgagacaataaccctgataaatgcttcaataatattgaaaaaggaagagtatgagtattcaacatttccgtgtcgcccttattcccttttttgcggcattttgccttcctgtttttgctcacccagaaacgctggtgaaagtaaaagatgctgaagatcagttgggtgcacgagtgggttacatcgaactggatctcaacagcggtaagatccttgagagttttcgccccgaagaacgttttccaatgatgagcacttttaaagttctgctatgtggcgcggtattatcccgtattgacgccgggcaagagcaactcggtcgccgcatacactattctcagaatgacttggttgagtactcaccagtcacagaaaagcatcttacggatggcatgacagtaagagaattatgcagtgctgccataaccatgagtgataacactgcggccaacttacttctgacaacgatcggaggaccgaaggagctaaccgcttttttgcacaacatgggggatcatgtaactcgccttgatcgttgggaaccggagctgaatgaagccataccaaacgacgagcgtgacaccacgatgcctgtagcaatggcaacaacgttgcgcaaactattaactggcgaactacttactctagcttcccggcaacaattaatagactggatggaggcggataaagttgcaggaccacttctgcgctcggcccttccggctggctggtttattgctgataaatctggagccggtgagcgtgggtctcgcggtatcattgcagcactggggccagatggtaagccctcccgtatcgtagttatctacacgacggggagtcaggcaactatggatgaacgaaatagacagatcgctgagataggtgcctcactgattaagcattggtaactgtcagaccaagtttactcatatatactttagattgatttaaaacttcatttttaatttaaaaggatctaggtgaagatcctttttgataatctcatgaccaaaatcccttaacgtgagttttcgttccactgagcgtcagaccccgtagaaaagatcaaaggatcttc ttgagatcctttttttctgcgcgtaatctgctgcttgcaaacaaaaaaaccaccgctaccagcggtggtttgtttgccggatcaagagctaccaactctttttccgaaggtaactggcttcagcagagcgcagataccaaatactgtccttctagtgtagccgtagttaggccaccacttcaagaactctgtagcaccgcctacatacctcgctctgctaatcctgttaccagtggctgctgccagtggcgataagtcgtgtcttaccgggttggactcaagacgatagttaccggataaggcgcagcggtcgggctgaacggggggttcgtgcacacagcccagcttggagcgaacgacctacaccgaactgagatacctacagcgtgagctatgagaaagcgccacgcttcccgaagggagaaaggcggacaggtatccggtaagcggcagggtcggaacaggagagcgcacgagggagcttccagggggaaacgcctggtatctttatagtcctgtcgggtttcgccacctctgacttgagcgtcgatttttgtgatgctcgtcaggggggcggagcctatggaaaaacgccagcaacgcggcctttttacggttcctggccttttgctggccttttgctcacatgttctttcctgcgttatcccctgattctgtggataaccgtattaccgcctttgagtgagctgataccgctcgccgcagccgaacgaccgagcgcagcgagtcagtgagcgaggaagcggaagagcgcccaatacgcaaaccgcctctccccgcgcgttggccgattcattaatgcagctggcacgacaggtttcccgactggaaagcgggcagtgagcgcaacgcaattaatgtgagttacctcactcattaggcaccccaggctttacactttatgcttccggctcctatgttgtgtggaattgtgagcggataacaatttcacacaggaaacagctatgaccatgattacgccaagcgcgcaattaaccctcactaaagggaacaaaagctgGAGCTC-3’  SNR52 promoter-promoter-[Insert 20 nucleotide gRNA protospacer]- structural crRNA-SUP4 terminator-f1 ori-CYC1 terminator-hphNT1 cassette-TEF1p promoter-2μ ori-AmpR promoter-AmpR cassette-ori |
| --- | --- |
| Cas9 in pRS415 | 5’-CATAGCTTCAAAATGTTTCTACTCCTTTTTTACTCTTCCAGATT  TTCTCGGACTCCGCGCATCGCCGTACCACTTCAAAACACCCAAGCACAGCATACTAAATTTCCCCTCTTTCTTCCTCTAGGGTGTCGTTAATTACCCGTACTAAAGGTTTGGAAAAGAAAAAAGAGACCGCCTCGTTTCTTTTTCTTCGTCGAAAAAGGCAATAAAAATTTTTATCACGTTTCTTTTTCTTGAAAATTTTTTTTTTGATTTTTTTCTCTTTCGATGACCTCCCATTGATATTTAAGTTAATAAACGGTCTTCAATTTCTCAAGTTTCAGTTTCATTTTTCTTGTTCTATTACAACTTTTTTTACTTCTTGCTCATTAGAAAGAAAGCATAGCAATCTAATCTAAGTTTTCTAGAACTAGTGGATCCCCCGGGaaaaATGGACAAGAAGTACTCCATTGGGCTCGATATCGGCACAAACAGCGTCGGCTGGGCCGTCATTACGGACGAGTACAAGGTGCCGAGCAAAAAATTCAAAGTTCTGGGCAATACCGATCGCCACAGCATAAAGAAGAACCTCATTGGCGCCCTCCTGTTCGACTCCGGGGAGACGGCCGAAGCCACGCGGCTCAAAAGAACAGCACGGCGCAGATATACCCGCAGAAAGAATCGGATCTGCTACCTGCAGGAGATCTTTAGTAATGAGATGGCTAAGGTGGATGACTCTTTCTTCCATAGGCTGGAGGAGTCCTTTTTGGTGGAGGAGGATAAAAAGCACGAGCGCCACCCAATCTTTGGCAATATCGTGGACGAGGTGGCGTACCATGAAAAGTACCCAACCATATATCATCTGAGGAAGAAGCTTGTAGACAGTACTGATAAGGCTGACTTGCGGTTGATCTATCTCGCGCTGGCGCATATGATCAAATTTCGGGGACACTTCCTCATCGAGGGGGACCTGAACCCAGACAACAGCGATGTCGACAAACTCTTTATCCAACTGGTTCAGACTTACAATCAGCTTTTCGAAGAGAACCCGATCAACGCATCCGGAGTTGACGCCAAAGCAATCCTGAGCGCTAGGCTGTCCAAATCCCGGCGGCTCGAAAACCTCATCGCACAGCTCCCTGGGGAGAAGAAGAACGGCCTGTTTGGTAATCTTATCGCCCTGTCACTCGGGCTGACCCCCAACTTTAAATCTAACTTCGACCTGGCCGAAGATGCCAAGCTTCAACTGAGCAAAGACACCTACGATGATGATCTCGACAATCTGCTGGCCCAGATCGGCGACCAGTACGCAGACCTTTTTTTGGCGGCAAAGAACCTGTCAGACGCCATTCTGCTGAGTGATATTCTGCGAGTGAACACGGAGATCACCAAAGCTCCGCTGAGCGCTAGTATGATCAAGCGCTATGATGAGCACCACCAAGACTTGACTTTGCTGAAGGCCCTTGTCAGACAGCAACTGCCTGAGAAGTACAAGGAAATTTTCTTCGATCAGTCTAAAAATGGCTACGCCGGATACATTGACGGCGGAGCAAGCCAGGAGGAATTTTACAAATTTATTAAGCCCATCTTGGAAAAAATGGACGGCACCGAGGAGCTGCTGGTAAAGCTTAACAGAGAAGATCTGTTGCGCAAACAGCGCACTTTCGACAATGGAAGCATCCCCCACCAGATTCACCTGGGCGAACTGCACGCTATCCTCAGGCGGCAAGAGGATTTCTACCCCTTTTTGAAAGATAACAGGGAAAAGATTGAGAAAATCCTCACATTTCGGATACCCTACTATGTAGGCCCCCTCGCCCGGGGAAATTCCAGATTCGCGTGGATGACTCGCAAATCAGAAGAGACCATCACTCCCTGGAACTTCGAGGAAGTCGTGGATAAGGGGGCCTCTGCCCAGTCCTTCATCGAAAGGATGACTAACTTTGATAAAAATCTGCCTAACGAAAAGGTGCTTCCTAAACACTCTCTGCTGTACGAGTACTTCACAGTTTATAACGAGCTCACCAAGGTCAAATACGTCACAGAAGGGATGAGAAAGCCAGCATTCCTGTCTGGAGAGCAGAAGAAAGCTATCGTGGACCTCCTCTTCAAGACGAACCGGAAAGTTACCGTGAAACAGCTCAAAGAAGACTATTTCAAAAAGATTGAATGTTTCGACTCTGTTGAAATCAGCGGAGTGGAGGATCGCTTCAACGCATCCCTGGGAACGTATCACGATCTCCTGAAAATCATTAAAGACAAGGACTTCCTGGACAATGAGGAGAACGAGGACATTCTTGAGGACATTGTCCTCACCCTTACGTTGTTTGAAGATAGGGAGATGATTGAAGAACGCTTGAAAACTTACGCTCATCTCTTCGACGACAAAGTCATGAAACAGCTCAAGAGGCGCCGATATACAGGATGGGGGCGGCTGTCAAGAAAACTGATCAATGGGATCCGAGACAAGCAGAGTGGAAAGACAATCCTGGATTTTCTTAAGTCCGATGGATTTGCCAACCGGAACTTCATGCAGTTGATCCATGATGACTCTCTCACCTTTAAGGAGGACATCCAGAAAGCACAAGTTTCTGGCCAGGGGGACAGTCTTCACGAGCACATCGCTAATCTTGCAGGTAGCCCAGCTATCAAAAAGGGAATACTGCAGACCGTTAAGGTCGTGGATGAACTCGTCAAAGTAATGGGAAGGCATAAGCCCGAGAATATCGTTATCGAGATGGCCCGAGAGAACCAAACTACCCAGAAGGGACAGAAGAACAGTAGGGAAAGGATGAAGAGGATTGAAGAGGGTATAAAAGAACTGGGGTCCCAAATCCTTAAGGAACACCCAGTTGAAAACACCCAGCTTCAGAATGAGAAGCTCTACCTGTACTACCTGCAGAACGGCAGGGACATGTACGTGGATCAGGAACTGGACATCAATCGGCTCTCCGACTACGACGTGGATCATATCGTGCCCCAGTCTTTTCTCAAAGATGATTCTATTGATAATAAAGTGTTGACAAGATCCGATAAAAATAGAGGGAAGAGTGATAACGTCCCCTCAGAAGAAGTTGTCAAGAAAATGAAAAATTATTGGCGGCAGCTGCTGAACGCCAAACTGATCACACAACGGAAGTTCGATAATCTGACTAAGGCTGAACGAGGTGGCCTGTCTGAGTTGGATAAAGCCGGCTTCATCAAAAGGCAGCTTGTTGAGACACGCCAGATCACCAAGCACGTGGCCCAAATTCTCGATTCACGCATGAACACCAAGTACGATGAAAATGACAAACTGATTCGAGAGGTGAAAGTTATTACTCTGAAGTCTAAGCTGGTCTCAGATTTCAGAAAGGACTTTCAGTTTTATAAGGTGAGAGAGATCAACAATTACCACCATGCGCATGATGCCTACCTGAATGCAGTGGTAGGCACTGCACTTATCAAAAAATATCCCAAGCTTGAATCTGAATTTGTTTACGGAGACTATAAAGTGTACGATGTTAGGAAAATGATCGCAAAGTCTGAGCAGGAAATAGGCAAGGCCACCGCTAAGTACTTCTTTTACAGCAATATTATGAATTTTTTCAAGACCGAGATTACACTGGCCAATGGAGAGATTCGGAAGCGACCACTTATCGAAACAAACGGAGAAACAGGAGAAATCGTGTGGGACAAGGGTAGGGATTTCGCGACAGTCCGGAAGGTCCTGTCCATGCCGCAGGTGAACATCGTTAAAAAGACCGAAGTACAGACCGGAGGCTTCTCCAAGGAAAGTATCCTCCCGAAAAGGAACAGCGACAAGCTGATCGCACGCAAAAAAGATTGGGACCCCAAGAAATACGGCGGATTCGATTCTCCTACAGTCGCTTACAGTGTACTGGTTGTGGCCAAAGTGGAGAAAGGGAAGTCTAAAAAACTCAAAAGCGTCAAGGAACTGCTGGGCATCACAATCATGGAGCGATCAAGCTTCGAAAAAAACCCCATCGACTTTCTCGAGGCGAAAGGATATAAAGAGGTCAAAAAAGACCTCATCATTAAGCTTCCCAAGTACTCTCTCTTTGAGCTTGAAAACGGCCGGAAACGAATGCTCGCTAGTGCGGGCGAGCTGCAGAAAGGTAACGAGCTGGCACTGCCCTCTAAATACGTTAATTTCTTGTATCTGGCCAGCCACTATGAAAAGCTCAAAGGGTCTCCCGAAGATAATGAGCAGAAGCAGCTGTTCGTGGAACAACACAAACACTACCTTGATGAGATCATCGAGCAAATAAGCGAATTCTCCAAAAGAGTGATCCTCGCCGACGCTAACCTCGATAAGGTGCTTTCTGCTTACAATAAGCACAGGGATAAGCCCATCAGGGAGCAGGCAGAAAACATTATCCACTTGTTTACTCTGACCAACTTGGGCGCGCCTGCAGCCTTCAAGTACTTCGACACCACCATAGACAGAAAGCGGTACACCTCTACAAAGGAGGTCCTGGACGCCACACTGATTCATCAGTCAATTACGGGGCTCTATGAAACAAGAATCGACCTCTCTCAGCTCGGTGGAGACAGCAGGGCTGACCCCAAGAAGAAGAGGAAGGTGTGATCTCTTCTCGAGTCATGTAATTAGTTATGTCACGCTTACATTCACGCCCTCCCCCCACATCCGCTCTAACCGAAAAGGAAGGAGTTAGACAACCTGAAGTCTAGGTCCCTATTTATTTTTTTATAGTTATGTTAGTATTAAGAACGTTATTTATATTTCAAATTTTTCTTTTTTTTCTGTACAGACGCGTGTACGCATGTAACATTATACTGAAAACCTTGCTTGAGAAGGTTTTGGGACGCTCGAAGGCTTTAATTTGCGGCCGGTACccaattcgccctatagtgagtcgtattacgcgcgctcactggccgtcgttttacaacgtcgtgactgggaaaaccctggcgttacccaacttaatcgccttgcagcacatccccctttcgccagctggcgtaatagcgaagaggcccgcaccgatcgcccttcccaacagttgcgcagcctgaatggcgaatggcgcgacgcgccctgtagcggcgcattaagcgcggcgggtgtggtggttacgcgcagcgtgaccgctacacttgccagcgccctagcgcccgctcctttcgctttcttcccttcctttctcgccacgttcgccggctttccccgtcaagctctaaatcgggggctccctttagggttccgatttagtgctttacggcacctcgaccccaaaaaacttgattagggtgatggttcacgtagtgggccatcgccctgatagacggtttttcgccctttgacgttggagtccacgttctttaatagtggactcttgttccaaactggaacaacactcaaccctatctcggtctattcttttgatttataagggattttgccgatttcggcctattggttaaaaaatgagctgatttaacaaaaatttaacgcgaattttaacaaaatattaacgtttacaatttcctgatgcggtattttctccttacgcatctgtgcggtatttcacaccgcatatcgacggtcgaggagaacttctagtatatccacatacctaatattattgccttattaaaaatggaatcccaacaattacatcaaaatccacattctcttcaaaatcaattgtcctgtacttccttgttcatgtgtgttcaaaaacgttatatttataggataattatactctatttctcaacaagtaattggttgtttggccgagcggtctaaggcgcctgattcaagaaatatcttgaccgcagttaactgtgggaatactcaggtatcgtaagatgcaagagttcgaatctcttagcaaccattatttttttcctcaacataacgagaacacacaggggcgctatcgcacagaatcaaattcgatgactggaaattttttgttaatttcagaggtcgcctgacgcatatacctttttcaactgaaaaattgggagaaaaaggaaaggtgagaggccggaaccggcttttcatatagaatagagaagcgttcatgactaaatgcttgcatcacaatacttgaagttgacaatattatttaaggacctattgttttttccaataggtggttagcaatcgtcttactttctaacttttcttaccttttacatttcagcaatatatatatatatttcaaggatataccattctaatgtctgcccctatgtctgcccctaagaagatcgtcgttttgccaggtgaccacgttggtcaagaaatcacagccgaagccattaaggttcttaaagctatttctgatgttcgttccaatgtcaagttcgatttcgaaaatcatttaattggtggtgctgctatcgatgctacaggtgtcccacttccagatgaggcgctggaagcctccaagaaggttgatgccgttttgttaggtgctgtgggtggtcctaaatggggtaccggtagtgttagacctgaacaaggtttactaaaaatccgtaaagaacttcaattgtacgccaacttaagaccatgtaactttgcatccgactctcttttagacttatctccaatcaagccacaatttgctaaaggtactgacttcgttgttgtcagagaattagtgggaggtatttactttggtaagagaaaggaagacgatggtgatggtgtcgcttgggatagtgaacaatacaccgttccagaagtgcaaagaatcacaagaatggccgctttcatggccctacaacatgagccaccattgcctatttggtccttggataaagctaatgttttggcctcttcaagattatggagaaaaactgtggaggaaaccatcaagaacgaattccctacattgaaggttcaacatcaattgattgattctgccgccatgatcctagttaagaacccaacccacctaaatggtattataatcaccagcaacatgtttggtgatatcatctccgatgaagcctccgttatcccaggttccttgggtttgttgccatctgcgtccttggcctctttgccagacaagaacaccgcatttggtttgtacgaaccatgccacggttctgctccagatttgccaaagaataaggttgaccctatcgccactatcttgtctgctgcaatgatgttgaaattgtcattgaacttgcctgaagaaggtaaggccattgaagatgcagttaaaaaggttttggatgcaggtatcagaactggtgatttaggtggttccaacagtaccaccgaagtcggtgatgctgtcgccgaagaagttaagaaaatccttgcttaaaaagattctctttttttatgatatttgtacataaactttataaatgaaattcataatagaaacgacacgaaattacaaaatggaatatgttcatagggtagacgaaactatatacgcaatctacatacatttatcaagaaggagaaaaaggaggatagtaaaggaatacaggtaagcaaattgatactaatggctcaacgtgataaggaaaaagaattgcactttaacattaatattgacaaggaggagggcaccacacaaaaagttaggtgtaacagaaaatcatgaaactacgattcctaatttgatattggaggattttctctaaaaaaaaaaaaatacaacaaataaaaaacactcaatgacctgaccatttgatggagtttaagtcaataccttcttgaaccatttcccataatggtgaaagttccctcaagaattttactctgtcagaaacggccttacgacgtagtcgatatggtgcactctcagtacaatctgctctgatgccgcatagttaagccagccccgacacccgccaacacccgctgacgcgccctgacgggcttgtctgctcccggcatccgcttacagacaagctgtgacaaagggcctcgtgatacgcctatttttataggttaatgtcatgataataatggtttcttaggacggatcgcttgcctgtaacttacacgcgcctcgtatcttttaatgatggaataatttgggaatttactctgtgtttatttatttttatgttttgtatttggattttagaaagtaaataaagaaggtagaagagttacggaatgaagaaaaaaaaataaacaaaggtttaaaaaatttcaacaaaaagcgtactttacatatatatttattagacaagaaaagcagattaaatagatatacattcgattaacgataagtaaaatgtaaaatcacaggattttcgtgtgtggtcttctacacagacaagatgaaacaattcggcattaatacctgagagcaggaagagcaagataaaaggtagtatttgttggcgatccccctagagtcttttacatcttcggaaaacaaaaactattttttctttaatttctttttttactttctatttttaatttatatatttatattaaaaaatttaaattataattatttttatagcacgtgatgaaaaggacccaggtggcacttttcggggaaatgtgcgcggaacccctatttgtttatttttctaaatacattcaaatatgtatccgctcatgagacaataaccctgataaatgcttcaataatattgaaaaaggaagagtatgagtattcaacatttccgtgtcgcccttattcccttttttgcggcattttgccttcctgtttttgctcacccagaaacgctggtgaaagtaaaagatgctgaagatcagttgggtgcacgagtgggttacatcgaactggatctcaacagcggtaagatccttgagagttttcgccccgaagaacgttttccaatgatgagcacttttaaagttctgctatgtggcgcggtattatcccgtattgacgccgggcaagagcaactcggtcgccgcatacactattctcagaatgacttggttgagtactcaccagtcacagaaaagcatcttacggatggcatgacagtaagagaattatgcagtgctgccataaccatgagtgataacactgcggccaacttacttctgacaacgatcggaggaccgaaggagctaaccgcttttttgcacaacatgggggatcatgtaactcgccttgatcgttgggaaccggagctgaatgaagccataccaaacgacgagcgtgacaccacgatgcctgtagcaatggcaacaacgttgcgcaaactattaactggcgaactacttactctagcttcccggcaacaattaatagactggatggaggcggataaagttgcaggaccacttctgcgctcggcccttccggctggctggtttattgctgataaatctggagccggtgagcgtggatctcgcggtatcattgcagcactggggccagatggtaagccctcccgtatcgtagttatctacacgacggggagtcaggcaactatggatgaacgaaatagacagatcgctgagataggtgcctcactgattaagcattggtaactgtcagaccaagtttactcatatatactttagattgatttaaaacttcatttttaatttaaaaggatctaggtgaagatcctttttgataatctcatgaccaaaatcccttaacgtgagttttcgttccactgagcgtcagaccccgtagaaaagatcaaaggatcttcttgagatcctttttttctgcgcgtaatctgctgcttgcaaacaaaaaaaccaccgctaccagcggtggtttgtttgccggatcaagagctaccaactctttttccgaaggtaactggcttcagcagagcgcagataccaaatactgtccttctagtgtagccgtagttaggccaccacttcaagaactctgtagcaccgcctacatacctcgctctgctaatcctgttaccagtggctgctgccagtggcgataagtcgtgtcttaccgggttggactcaagacgatagttaccggataaggcgcagcggtcgggctgaacggggggttcgtgcacacagcccagcttggagcgaacgacctacaccgaactgagatacctacagcgtgagctatgagaaagcgccacgcttcccgaagggagaaaggcggacaggtatccggtaagcggcagggtcggaacaggagagcgcacgagggagcttccagggggaaacgcctggtatctttatagtcctgtcgggtttcgccacctctgacttgagcgtcgatttttgtgatgctcgtcaggggggcggagcctatggaaaaacgccagcaacgcggcctttttacggttcctggccttttgctggccttttgctcacatgttctttcctgcgttatcccctgattctgtggataaccgtattaccgcctttgagtgagctgataccgctcgccgcagccgaacgaccgagcgcagcgagtcagtgagcgaggaagcggaagagcgcccaatacgcaaaccgcctctccccgcgcgttggccgattcattaatgcagctggcacgacaggtttcccgactggaaagcgggcagtgagcgcaacgcaattaatgtgagttacctcactcattaggcaccccaggctttacactttatgcttccggctcctatgttgtgtggaattgtgagcggataacaatttcacacaggaaacagctatgaccatgattacgccaagcgcgcaattaaccctcactaaagggaacaaaagctggAGCT-3’  TEF promoter-Cas9 cassette-CYC1 terminator-T7 promoter-f1 ori-pLEU2 guess promoter-LEU2 cassette-CEN/ARS- AmpR promoter-AmpR cassette- pBR322 ori |
| violacein pathway | 5’-catgcgactgggtgagcatatgttccgctgatgtgatgtgcaagataaacaagcaaggcagaaactaa  cttcttcttcatgtaataaacacaccccgcgtttatttacctatctctaaacttcaacaccttatatcataactaatatttcttgagataagcacactgcacccataccttccttaaaaacgtagcttccagtttttggtggttccggcttccttcccgattccgcccgctaaacgcatatttttgttgcctggtggcatttgcaaaatgcataacctatgcatttaaaagattatgtatgctcttctgacttttcgtgtgatgaggctcgtggaaaaaatgaataatttatgaatttgagaacaattttgtgttgttacggtattttactatggaataatcaatcaattgaggattttatgcaaatatcgtttgaatatttttccgaccctttgagtacttttcttcataattgcataatattgtccgctgcccctttttctgttagacggtgtcttgatctacttgctatcgttcaacaccaccttattttctaactattttttttttagctcatttgaatcagcttatggtgatggcacatttttgcataaacctagctgtcctcgttgaacataggaaaaaaaaatatataaacaaggctctttcactctccttgcaatcagatttgggtttgttccctttattttcatatttcttgtcatattcctttctcaattattattttctactcataacctcacgcaaaataacacagtcaaatcaatcaaaccttgcaaaatgaaacattcttccgatatctgcattgttggtgctggtatttctggtttgacgtgcgcaagccatctgctggacagcccggcatgccgtggtctgagcctgcgtatctttgacatgcagcaagaagccggtggccgtatccgcagcaaaatgctggatggtaaggcaagcattgaactgggcgcaggtcgctactcccctcagttgcacccgcatttccaaagcgcaatgcagcactatagccaaaagagcgaagtctatccgttcacccagttgaagttcaaatctcacgtgcagcaaaagctgaagcgcgccatgaatgaactgtccccgcgtctgaaagagcatggtaaagagagctttttgcagtttgtcagccgttatcaaggtcacgatagcgcggttggtatgatccgctctatgggttacgacgcactgttcctgccggatatcagcgcagaaatggcctacgacattgtgggtaagcacccggagatccagagcgtgacggacaacgacgcgaaccaatggtttgcagcggaaacgggctttgctggtctgattcagggcatcaaggctaaggttaaggcggcaggtgcgcgttttagcctgggttatcgtctgctgagcgtccgtaccgacggtgacggctacctgctgcaactggcaggtgacgacggctggaaactggagcaccgtacccgccatctgattctggcgattccgccgagcgcgatggcgggtttgaatgttgattttccagaagcctggtccggtgcgcgctatggcagcctgccgctgtttaagggctttctgacgtacggtgagccgtggtggttggactacaaactggacgatcaggtgctgattgttgacaacccgctgcgcaaaatctatttcaaaggcgataagtacctgttcttctataccgatagcgagatggcgaattactggcgcggttgtgtcgcggagggcgaggacggttacctggagcaaattcgcacccatttggctagcgcactgggtatcgtccgtgaacgtatcccgcaaccgctggcacacgttcacaagtattgggcgcacggcgttgagttttgccgtgattctgatattgaccacccgagcgcactgtctcatcgcgacagcggtatcatcgcgtgctccgatgcgtacacggagcattgtggttggatggagggcggtctgctgagcgcccgtgaggcaagccgtctgctgttgcagcgtatcgccgcgtaagtctgaagaatgaatgatttgatgatttctttttccctccatttttcttactgaatatatcaatgatatagacttgtatagtttattatttcaaattaagtagctatatatagtcaagataacgtttgtttgacacgattacattattcgtcgacatcttttttcagcctgtcgtggtagcaatttgaggagtattattaattgaataggttcattttgcgctcgcataaacagttttcgtcagggacagtatgttggaatgagtggtaattaatggtgacatgacatgttatagcaataaccttgatgtttacatcgtagtttaatgtacaccccgcgaattcgttcaagtaggagtgcaccaattgcaaagggaaaagctgaatgggcagttcgaatatattttagattcctgacttcaactcaagacgcacagatattataacatctgcataataggcatttgcaagaattactcgtgagtaaggaaagagtgaggaactatcgcatacctgcatttaaagatgccgatttgggcgcgaatcctttattttggcttcaccctcatactattatcagggccagaaaaaggaagtgtttccctccttcttgaattgatgttaccctcataaagcacgtggcctcttatcgagaaagaaattaccgtcgctcgtgatttgtttgcaaaaagaacaaaactgaaaaaacccagacacgctcgacttcctgtcttcctattgattgcagcttccaatttcgtcacacaacaaggtcctagcgacggctcacaggttttgtaacaagcaatcgaaggttctggaatggcgggaaagggtttagtaccacatgctatgatgcccactgtgatctccagagcaaagttcgttcgatcgtactgttactctctctctttcaaacagaattgtccgaatcgtgtgacaacaacagcctgttctcacacactcttttcttctaaccaagggggtggtttagtttagtagaacctcgtgaaacttacatttacatatatataaacttgcataaattggtcaatgcaagaaatacatatttggtcttttctaattcgtagtttttcaagttcttagatgctttctttttctcttttttacagatcatcaaggaagtaattatctactttttacaacaaatataaaacagtttacaaatgagcattctggatttcccgcgtatccacttccgtggctgggcccgtgtcaatgcgccgaccgcgaaccgcgatccgcacggccacatcgatatggccagcaataccgtggcgatggcgggtgagccgttcgacctggcacgccatcctacggagttccaccgtcacctgcgctccctgggtccgcgcttcggcttggatggtcgtgctgacccggaaggcccgttcagcctggccgagggctacaacgctgccggtaacaaccacttttcgtgggagagcgcaaccgttagccacgtgcaatgggatggcggtgaggcggatcgtggtgacggtctggtcggtgctcgtttggcactgtggggtcactacaatgattatctgcgtaccaccttcaatcgtgctcgttgggtcgacagcgacccgacgcgccgtgacgctgcacaaatctatgcgggccaattcaccattagcccggctggtgccggtccgggtacgccgtggctgtttacggcagacattgatgatagccatggtgcacgttggacgcgtggcggccacattgcagagcgtggcggccacttcttggatgaagagtttggtctggcacgcctgtttcagttctctgtgccgaaagatcacccacattttctgtttcacccgggtccgtttgattccgaggcctggcgtcgtctgcaattggctctggaggatgacgacgttctgggtctgaccgtgcaatatgcgttgttcaatatgagcaccccgcctcagccgaacagcccggtttttcacgatatggtcggtgttgtcggtctgtggcgtcgtggtgaactggcgagctacccggctggtcgtctgctgcgtccgcgtcaaccgggtctgggtgacctgaccctgcgcgtcaacggtggtcgcgttgcgctgaatttggcgtgtgccattccgttcagcactcgtgccgcgcagccaagcgcaccggaccgcctgaccccggacctgggtgccaaactgccgctgggcgatctgctgctgcgtgatgaggacggcgcactgttggcacgtgtgccgcaggctctgtaccaagactattggacgaatcacggtattgtggacctgccgctgctgcgcgaaccgcgtggtagcttgaccctgagcagcgaactggcggagtggcgtgagcaagactgggtcacccaaagcgacgcgtctaacctgtacctggaggcaccggatcgccgtcacggtcgctttttccctgagagcatcgcgctgcgcagctactttcgcggtgaagcgcgtgcgcgtccggatatcccgcatcgtatcgagggcatgggcctggtcggcgtcgaatctcgtcaggatggcgacgctgcggaatggcgtctgacgggtctgcgtccgggtccggcacgcattgttctggacgatggtgccgaggcgatccctctgcgtgttctgcctgacgattgggcgctggatgacgcgaccgtcgaagaagtggattacgcctttttgtaccgccacgttatggcgtattacgagctggtgtatccattcatgagcgacaaggtgttttccctggctgatcgttgcaaatgtgaaacgtacgcacgtctgatgtggcagatgtgtgatccgcagaaccgcaacaagtcctattacatgccgagcacccgcgaactgtcggcaccgaaagctcgtttgttcttgaagtatctggcccacgtggaaggccaggcacgcctgcaagcacctccgccagcgggtccggcacgcattgaatctaaagcccagttggcggcagagctgcgtaaagccgtcgacctggagctgtctgtgatgctgcaatacctgtacgcggcgtatagcattccgaactatgcacagggccaacaacgtgttcgtgacggtgcgtggaccgccgagcagctgcaactggcgtgcggtagcggtgaccgtcgccgtgatggcggtattcgtgcagcactgctggaaattgctcatgaagaaatgattcattacctggtcgttaacaacctgctgatggccctgggcgagccgttctacgcgggtgtcccgctgatgggcgaagcggcacgtcaggcgtttggcctggacaccgagttcgctctggaaccgtttagcgaaagcacgctggcacgttttgttcgtctggaatggccgcactttatcccagcaccgggcaaatccatcgcggactgctatgccgccattcgtcaggcgtttttggatctgccggacttgtttggtggcgaggcaggtaagcgtggcggtgaacaccacctgttcctgaatgagctgaccaaccgtgcgcatccgggttatcaactggaagttttcgatcgcgactcggcgctgtttggtattgcatttgtgaccgatcagggcgaaggtggcgctctggacagcccgcactacgaacatagccattttcaacgtctgcgtgaaatgagcgcgcgtatcatggctcaaagcgcaccgttcgaaccggcgctgccggcgttgcgtaatccggttctggatgagagcccgggttgccaacgtgtcgcagacggtcgtgcgcgtgcgctgatggcattgtaccaaggcgtttatgagctgatgtttgcgatgatggcgcagcacttcgccgtgaaaccgctgggtagcttgcgtcgcagccgcctgatgaacgcagcaatcgatctgatgaccggtctgttgcgtccgctgagctgcgcgctgatgaacctgccaagcggcatcgccggtcgcacggccggtccgccgctgccgggtccggttgacacccgtagctatgacgactacgcgctgggctgtcgcatgctggcacgccgttgcgagcgtctgctggagcaggcgagcatgctggaaccgggttggctgccggatgcgcagatggagctgctggatttctatcgtcgccaaatgctggacttggcgtgcggcaaactgagccgcgaggcctaagtgaatttactttaaatcttgcatttaaataaattttctttttatagctttatgacttagtttcaatttatatactattttaatgacattttcgattcattgattgaaagctttgtgttttttcttgatgcgctattgcattgttcttgtctttttcgccacatgtaatatctgtagtagatacctgatacattgtggatgctgagtgaaattttagttaataatggaggcgctcttaataattttggggatattggcttttttttttaaagtttacaaatgaattttttccgccaggataacgattctgaagttactcttagcgttcctatcggtacagccatcaaatcatgcctataaatcatgcctatatttgcgtgcagtcagtatcatctacatgaaaaaaactcccgcaatttcttatagaatacgttgaaaattaaatgtacgcgccaagataagataacatatatctagatgcagtaatatacacagattccggtgggtgtgggtgtattggattataggaagccacgcgctcaacctggaattacaggaagctggtaattttttgggtttgcaatcatcaccatctgcacgttgttataatgtcccgtgtctatatatatccattgacggtattctatttttttgctattgaaatgagcgttttttgttactacaattggttttacagacggaattttccctatttgtttcgtcccatttttccttttctcattgttctcatatcttaaaaaggtcctttcttcataatcaatgctttcttttacttaatattttacttgcattcagtgaattttaatacatattcctctagtcttgcaaaatcgatttagaatcaagataccagcctaaaactgcccaaaatggagaaccgtgagccaccactgttgccagcccgttggagcagcgcctatgtctcttattggagcccgatgctgccggatgaccagctgaccagcggctattgctggttcgactatgaacgtgacatctgtcgtattgacggcctgttcaatccgtggagcgagcgtgatactggttatcgcctgtggatgtcggaggttggtaatgcggccagcggccgtacctggaaacaaaaagtcgcctatggtcgtgagcgtaccgccctgggtgaacagctgtgtgagcgtccgctggatgatgagactggcccttttgccgaattgttcctgccacgcgatgtcctgcgccgtctgggtgcccgtcacattggccgtcgcgtggttctgggtcgcgaagcggacggttggcgttaccagcgcccaggtaaaggtccgagcaccctgtacctggatgcggcgagcggcactccactgcgcatggtcaccggcgatgaagcgtcgcgtgcaagcctgcgtgattttccgaatgtgagcgaggcggagatcccggacgcggttttcgcggccaagcgctaagttaattcaaattaattgatatagttttttaatgagtattgaatctgtttagaaataatggaatattatttttatttatttatttatattattggtcggctcttttcttctgaaggtcaatgacaaaatgatatgaaggaaataatgatttctaaaattttacaacgtaagatatttttacaaaagcctagctcatcttttatatctaggaacccatcaggttggtggaagattacccgttctaagacttttcagcttcctctattgatgttacacctggacaccccttttctggcatccagtttttaatcttcagtggcatgtgagattctccgaaattaattaaagcaatcacacaattctctcggataccacctcggttgaaactgacaggtggtttgttacgcatgctaatgcaaaggagcctatatacctttggctcggctgctgtaacagggaatataaagggcagcataatttaggagtttagtgaacttgcaacatttactattttcccttcttacgtaaatatttttctttttaattctaaatcaatctttttcaattttttgtttgtattcttttcttgcttaaatctataactacaaaaaacacatacataaactaaaacaaaatgaagattctggtcattggtgctggtccagctggtctggttttcgcatcccaactgaagcaggcacgccctttgtgggccattgacatcgtggagaagaatgacgagcaagaagtgctgggctggggtgtcgtgctgcctggccgtccgggtcagcacccggcgaacccgctgtcctatctggatgcaccggagcgtctgaatccgcaatttctggaggacttcaaactggtgcatcataatgagccgtccttgatgtccacgggcgttttgttgtgcggcgtggagcgtcgcggtctggttcacgcgctgcgcgataagtgccgcagccaaggcattgctattcgtttcgaaagcccgttgctggaacacggtgagctgccgctggcggactatgatctggtggtcctggctaatggtgttaatcacaaaaccgcgcatttcaccgaggctctggtcccgcaggtggactacggccgcaataagtacatttggtatggcactagccagctgttcgatcagatgaatctggtttttcgtacccatggtaaagatatctttatcgcgcatgcctataagtatagcgataccatgagcacgttcattgtcgaatgtagcgaagagacttacgcacgcgcacgcctgggcgaaatgtccgaagaggcgagcgcagaatacgttgctaaggtgttccaggccgagctgggtggtcacggcctggtgagccagccgggtctgggttggcgtaacttcatgacgttgtctcatgaccgttgtcatgatggtaagttggttctgctgggtgacgcgctgcaaagcggtcactttagcatcggccacggcaccacgatggccgtggtggtggcgcagctgctggttaaagcgctgtgtaccgaagatggtgtgcctgccgcgctgaaacgtttcgaagagcgtgccctgccgctggtgcagttgttccgtggccacgcagacaacagccgcgtttggttcgaaaccgtcgaagagcgcatgcacctgtcctcggcggaatttgtgcaaagcttcgacgcacgccgcaaaagcctgccgccgatgccggaagcactggcgcagaatctgcgttatgctttgcagcgctgataaattgaattgaattgaaatcgatagatcaatttttttcttttctctttccccatcctttacgctaaaataatagtttattttattttttgaatattttttatttatatacgtatatatagactattatttatcttttaatgattattaagatttttattaaaaaaaaattcgctcctcttttaatgcctttatgcagtttttttttctcgatatttctatgttcgggttcagcgtattttaagtttaataactcgaaaattctgcgttcgtttgtatatgctcatttacactctatatcaccatatggaggataagttgggctgagcttctgatccaatttattctatccattagttgctgatatgtcccaccagccaacacttgatagtatctactcgccattcacttccagcagcgccagtagggttgttgagcttagtaaaaatgtgcgcaccacaagcctacatgactccacgtcacatgaaaccacaccgtggggccttgttgcgctaggaataggatatgcgacgaagacgcttctgcttagtaaccacaccacattttcagggggtcgatctgcttgcttcctttactgtcacgagcggcccataatcgcgctttttttttaaaaggcgcgagacagcaaacaggaagctcgggtttcaaccttcggagtggtcgcagatctggagactggatctttacaatacagtaaggcaagccaccatctgcttcttaggtgcatgcgacggtatccacgtgcagaacaacatagtctgaagaagggggggaggagcatgttcattctctgtagcagtaagagcttggtgataatgaccaaaactggagtctcgaaatcatataaatagacaatatattttcacacaatgagatttgtagtacagttctattctctctcttgcataaataagaaattcatcaagaacttggtttgatatttcaccaacacacacaaaaaacagtacttcactaaatttacacacaaaacaaaggctacaaaatgaaacgtgcgattatcgttggtggcggcctggcgggtggcctgaccgcgatctacctggcgaagcgtggctacgaagtgcacgtcgtggagaagcgtggtgatcctctgcgcgatctgagctcttacgtggacgttgttagcagccgtgcgatcggcgtgagcatgaccgttcgtggtatcaagagcgttttggctgcgggcattccgcgtgcagagctggatgcgtgtggcgaaccgatcgtggcaatggctttctccgtgggtggtcagtatcgcatgcgcgaactgaagccgttggaggatttccgtccgctgagcttgaaccgtgcggcgtttcaaaagctgctgaacaaatacgcgaacctggcaggcgttcgttactactttgagcataagtgcctggatgttgacctggatggtaagagcgtgttgattcagggcaaagatggtcagccgcagcgtctgcaaggtgacatgattatcggtgcggatggcgcccacagcgccgtccgtcaggcgatgcagagcggcctgcgtcgtttcgagttccagcaaacgttcttccgccatggctacaaaaccctggttttgccggacgcgcaagcactgggttaccgtaaagacacgctgtactttttcggcatggattccggtggcctgttcgcgggtcgtgcggctacgatcccagatggtagcgtcagcatcgccgtttgcctgccgtactcgggtagcccttccctgacgaccaccgacgaaccgacgatgcgtgcgttcttcgatcgttacttcggtggcctgccgcgtgacgcgcgtgacgaaatgctgcgtcagtttctggcgaagccgagcaacgacctgattaacgtgcgctctagcacctttcactataagggtaatgtgctgttgctgggtgatgctgcgcatgcgactgcgccgttcctgggtcagggtatgaacatggcgctggaggacgcccgcacgtttgtcgagctgctggaccgccaccagggcgaccaagacaaagcctttccggagttcacggagctgcgcaaagtccaggcagacgcaatgcaagacatggctcgcgccaactatgacgttttgagctgctcgaacccgatctttttcatgcgtgcgcgttacacgcgttacatgcattccaagtttccgggcctgtatccgccggatatggccgagaaactgtactttacgagcgagccgtacgatcgtctgcaacaaatccagcgtaaacagaatgtttggtacaagattggtcgcgtgaattaacatgtaattagttatgtcacgcttacattcacgccctccccccacatccgctctaaccgaaaaggaaggagttagacaacctgaagtctaggtccctatttatttttttatagttatgttagtattaagaacgttatttatatttcaaatttttcttttttttctgtacagacgcgtgtacgcatgtaacattatactgaaaaccttgcttgagaaggttttgggacgctcgaaggctttgcaccataccacagcttttcaattcaattcatcattttttttttattcttttttttgatttcggtttctttgaaatttttttgattcggtaatctccgaacagaaggaagaacgaaggaaggagcacagacttagattggtatatatacgcatatgtagtgttgaagaaacatgaaattgcccagtattcttaacccaactgcacagaacaaaaacctgcaggaaacgaagataaatcatgtcgaaagctacatataaggaacgtgctgctactcatcctagtcctgttgctgccaagctatttaatatcatgcacgaaaagcaaacaaacttgtgtgcttcattggatgttcgtaccaccaaggaattactggagttagttgaagcattaggtcccaaaatttgtttactaaaaacacatgtggatatcttgactgatttttccatggagggcacagttaagccgctaaaggcattatccgccaagtacaattttttactcttcgaagacagaaaatttgctgacattggtaatacagtcaaattgcagtactctgcgggtgtatacagaatagcagaatgggcagacattacgaatgcacacggtgtggtgggcccaggtattgttagcggtttgaagcaggcggcagaagaagtaacaaaggaacctagaggccttttgatgttagcagaattgtcatgcaagggctccctatctactggagaatatactaagggtactgttgacattgcgaagagcgacaaagattttgttatcggctttattgctcaaagagacatgggtggaagagatgaaggttacgattggttgattatgacacccggtgtgggtttagatgacaagggagacgcattgggtcaacagtatagaaccgtggatgatgtggtctctacaggatctgacattattattgttggaagaggactatttgcaaagggaagggatgctaaggtagagggtgaacgttacagaaaagcaggctgggaagcatatttgagaagatgcggccagcaaaactaaaaaactgtattataagtaaatgcatgtatactaaactcacaaattagagcttcaatttaattatatcagttattaccctatgcggtgtgaaataccgcacagatgcgtaaggagaaaataccgcatcagggaattccgaagggcaattctgcagatatccatcacactggcggccgctcgagcatgcatctagagggcccaattcgccctatagtgagtcgtattacaattcactggccgtcgttttacaacgtcgtgactgggaaaaccctggcgttacccaacttaatcgccttgcagcacatccccctttcgccagctggcgtaatagcgaagaggcccgcaccgatcgcccttcccaacagttgcgcagcctgaatggcgaatggacgcgccctgtagcggcgcattaagcgcggcgggtgtggtggttacgcgcagcgtgaccgctacacttgccagcgccctagcgcccgctcctttcgctttcttcccttcctttctcgccacgttcgccggctttccccgtcaagctctaaatcgggggctccctttagggttccgatttagtgctttacggcacctcgaccccaaaaaacttgattagggtgatggttcacgtagtgggccatcgccctgatagacggtttttcgccctttgacgttggagtccacgttctttaatagtggactcttgttccaaactggaacaacactcaaccctatctcggtctattcttttgatttataagggattttgccgatttcggcctattggttaaaaaatgagctgatttaacaaaaatttaacgcgaattttaacaaaattcagggcgcaagggctgctaaaggaagcggaacacgtagaaagccagtccgcagaaacggtgctgaccccggatgaatgtcagctactgggctatctggacaagggaaaacgcaagcgcaaagagaaagcaggtagcttgcagtgggcttacatggcgatagctagactgggcggttttatggacagcaagcgaaccggaattgccagctggggcgccctctggtaaggttgggaagccctgcaaagtaaactggatggctttcttgccgccaaggatctgatggcgcaggggatcaagatctgatcaagagacaggatgaggatcgtttcgcatgattgaacaagatggattgcacgcaggttctccggccgcttgggtggagaggctattcggctatgactgggcacaacagacaatcggctgctctgatgccgccgtgttccggctgtcagcgcaggggcgcccggttctttttgtcaagaccgacctgtccggtgccctgaatgaactgcaggacgaggcagcgcggctatcgtggctggccacgacgggcgttccttgcgcagctgtgctcgacgttgtcactgaagcgggaagggactggctgctattgggcgaagtgccggggcaggatctcctgtcatcccaccttgctcctgccgagaaagtatccatcatggctgatgcaatgcggcggctgcatacgcttgatccggctacctgcccattcgaccaccaagcgaaacatcgcatcgagcgagcacgtactcggatggaagccggtcttgtcgatcaggatgatctggacgaagagcatcaggggctcgcgccagccgaactgttcgccaggctcaaggcgcgcatgcccgacggcgaggatctcgtcgtgacccatggcgatgcctgcttgccgaatatcatggtggaaaatggccgcttttctggattcatcgactgtggccggctgggtgtggcggaccgctatcaggacatagcgttggctacccgtgatattgctgaagagcttggcggcgaatgggctgaccgcttcctcgtgctttacggtatcgccgctcccgattcgcagcgcatcgccttctatcgccttcttgacgagttcttctgaattgaaaaaggaagagtatgagtattcaacatttccgtgtcgcccttattcccttttttgcggcattttgccttcctgtttttgctcacccagaaacgctggtgaaagtaaaagatgctgaagatcagttgggtgcacgagtgggttacatcgaactggatctcaacagcggtaagatccttgagagttttcgccccgaagaacgttttccaatgatgagcacttttaaagttctgctatgtggcgcggtattatcccgtattgacgccgggcaagagcaactcggtcgccgcatacactattctcagaatgacttggttgagtactcaccagtcacagaaaagcatcttacggatggcatgacagtaagagaattatgcagtgctgccataaccatgagtgataacactgcggccaacttacttctgacaacgatcggaggaccgaaggagctaaccgcttttttgcacaacatgggggatcatgtaactcgccttgatcgttgggaaccggagctgaatgaagccataccaaacgacgagcgtgacaccacgatgcctgtagcaatggcaacaacgttgcgcaaactattaactggcgaactacttactctagcttcccggcaacaattaatagactggatggaggcggataaagttgcaggaccacttctgcgctcggcccttccggctggctggtttattgctgataaatctggagccggtgagcgtgggtctcgcggtatcattgcagcactggggccagatggtaagccctcccgtatcgtagttatctacacgacggggagtcaggcaactatggatgaacgaaatagacagatcgctgagataggtgcctcactgattaagcattggtaactgtcagaccaagtttactcatatatactttagattgatttaaaacttcatttttaatttaaaaggatctaggtgaagatcctttttgataatctcatgaccaaaatcccttaacgtgagttttcgttccactgagcgtcagaccccgtagaaaagatcaaaggatcttcttgagatcctttttttctgcgcgtaatctgctgcttgcaaacaaaaaaaccaccgctaccagcggtggtttgtttgccggatcaagagctaccaactctttttccgaaggtaactggcttcagcagagcgcagataccaaatactgttcttctagtgtagccgtagttaggccaccacttcaagaactctgtagcaccgcctacatacctcgctctgctaatcctgttaccagtggctgctgccagtggcgataagtcgtgtcttaccgggttggactcaagacgatagttaccggataaggcgcagcggtcgggctgaacggggggttcgtgcacacagcccagcttggagcgaacgacctacaccgaactgagatacctacagcgtgagctatgagaaagcgccacgcttcccgaagggagaaaggcggacaggtatccggtaagcggcagggtcggaacaggagagcgcacgagggagcttccagggggaaacgcctggtatctttatagtcctgtcgggtttcgccacctctgacttgagcgtcgatttttgtgatgctcgtcaggggggcggagcctatggaaaaacgccagcaacgcggcctttttacggttcctggccttttgctggccttttgctcacatgttctttcctgcgttatcccctgattctgtggataaccgtattaccgcctttgagtgagctgataccgctcgccgcagccgaacgaccgagcgcagcgagtcagtgagcgaggaagcggaagagcgcccaatacgcaaaccgcctctccccgcgcgttggccgattcattaatgcagctggcacgacaggtttcccgactggaaagcgggcagtgagcgcaacgcaattaatgtgagttagctcactcattaggcaccccaggctttacactttatgcttccggctcgtatgttgtgtggaattgtgagcggataacaatttcacacaggaaacagctatgaccatgattacgccaagcttggtaccgagctcggatccactagtaacggccgccagtgtgctggaattctgcagatatccatcacactggcggccgc-3’  PDC1 promoter-VIO-A cassette-GPM1 terminator-PGK1 promoter-VIO-B cassette-GPD terminator-PGI1 promoter-VIO-E cassette-FBA1 terminator-TPI1 promoter-VIO-D cassette-PGK1 terminator-TDH1 promoter-VIO-C cassette-CYC1 terminator-URA3 promoter-URA3 cassette-URA3 terminator-Kanamycin cassette-AmpR cassette |
| *KarI*Δ15 in pRS416 | 5’-GCACCATACCACAGCTTTTCAATTCAATTCATCATTTTTTTTTT  ATTCTTTTTTTTGATTTCGGTTTCTTTGAAATTTTTTTGATTCGGTAATCTCCGAACAGAAGGAAGAACGAAGGAAGGAGCACAGACTTAGATTGGTATATATACGCATATGTAGTGTTGAAGAAACATGAAATTGCCCAGTATTCTTAACCCAACTGCACAGAACAAAAACCTGCAGGAAACGAAGATAAATCATGTCGAAAGCTACATATAAGGAACGTGCTGCTACTCATCCTAGTCCTGTTGCTGCCAAGCTATTTAATATCATGCACGAAAAGCAAACAAACTTGTGTGCTTCATTGGATGTTCGTACCACCAAGGAATTACTGGAGTTAGTTGAAGCATTAGGTCCCAAAATTTGTTTACTAAAAACACATGTGGATATCTTGACTGATTTTTCCATGGAGGGCACAGTTAAGCCGCTAAAGGCATTATCCGCCAAGTACAATTTTTTACTCTTCGAAGACAGAAAATTTGCTGACATTGGTAATACAGTCAAATTGCAGTACTCTGCGGGTGTATACAGAATAGCAGAATGGGCAGACATTACGAATGCACACGGTGTGGTGGGCCCAGGTATTGTTAGCGGTTTGAAGCAGGCGGCAGAAGAAGTAACAAAGGAACCTAGAGGCCTTTTGATGTTAGCAGAATTGTCATGCAAGGGCTCCCTATCTACTGGAGAATATACTAAGGGTACTGTTGACATTGCGAAGAGCGACAAAGATTTTGTTATCGGCTTTATTGCTCAAAGAGACATGGGTGGAAGAGATGAAGGTTACGATTGGTTGATTATGACACCCGGTGTGGGTTTAGATGACAAGGGAGACGCATTGGGTCAACAGTATAGAACCGTGGATGATGTGGTCTCTACAGGATCTGACATTATTATTGTTGGAAGAGGACTATTTGCAAAGGGAAGGGATGCTAAGGTAGAGGGTGAACGTTACAGAAAAGCAGGCTGGGAAGCATATTTGAGAAGATGCGGCCAGCAAAACTAAAAAACTGTATTATAAGTAAATGCATGTATACTAAACTCACAAATTAGAGCTTCAATTTAATTATATCAGTTATTACCCTATGCGGTGTGAAATACCGCACAGATGCGTAAGGAGAAAATACCGCATCAGGAAATTGTAAACGTTAATATTTTGTTAAAATTCGCGTTAAATTTTTGTTAAATCAGCTCATTTTTTAACCAATAGGCCGAAATCGGCAAAATCCCTTATAAATCAAAAGAATAGACCGAGATAGGGTTGAGTGTTGTTCCAGTTTGGAACAAGAGTCCACTATTAAAGAACGTGGACTCCAACGTCAAAGGGCGAAAAACCGTCTATCAGGGCGATGGCCCACTACGTGAACCATCACCCTAATCAAGTTTTTTGGGGTCGAGGTGCCGTAAAGCACTAAATCGGAACCCTAAAGGGAGCCCCCGATTTAGAGCTTGACGGGGAAAGCCGGCGAACGTGGCGAGAAAGGAAGGGAAGAAAGCGAAAGGAGCGGGCGCTAGGGCGCTGGCAAGTGTAGCGGTCACGCTGCGCGTAACCACCACACCCGCCGCGCTTAATGCGCCGCTACAGGGCGCGTCGCGCCATTCGCCATTCAGGCTGCGCAACTGTTGGGAAGGGCGATCGGTGCGGGCCTCTTCGCTATTACGCCAGCTGGCGAAAGGGGGATGTGCTGCAAGGCGATTAAGTTGGGTAACGCCAGGGTTTTCCCAGTCACGACGTTGTAAAACGACGGCCAGTGAGCGCGCGTAATACGACTCACTATAGGGCGAATTGGGTACCGGGCCCCCCCTCGAGGTCGACGGTATCGATAAGCTTGATATCGAATTCCTGCAGCCCGGGGGATCCAGTTCGTACAGTATTCTCTTTCTCTGGGGTTTTTCCCTTTTATAGAAGTTGACTTGTTCTATAGTTGTTATATATGTATTGCTGGGTTTTTTAATCTATTTTGCGGAAGACGCCAAAAATAAGAAAAAAATTGGAAAGACACAAGAAAAAAGTAAGAATATCCACAGCTGAAAGATCTAACAGGCGTATTTTTATTTTCGAAGAAACGTCCTAAAAGGATCTGATTCAGCACATTGATAATAGCATAGGCATTTTTATCTTTCTTTTTTGAGTTCATGAATGAATGTAACTTCTCCAAAAGATGGGAATCACAGTTTCTCGAAGAAAAATAGATTTAATACAAATAAACCGCGATTCCACAAACTAAATGAGCAGGCGCAGAGTATTGCATCGCGTACCAAAAATATTAATTCTGATAGTGATAGAAGCAATGATACCATAAAACAAAACAACTACAATAAAAGGGAGACCGGATATAACCCTTTCTACAATGGATCAGGGATCAATTCACAAAGAAAATCTAGTGCAGCACTTCGGAAACAATTAGGAAAACCCCTACCGCTGCCGTATTTGAATAGCCCTAATAGTGATAGTACACCCACATTACAGAGAAAAGAAGAAGTATTCACAGACGAAGTGCTTCAAAAAAAGAGAGAATTGATTGAGTCTAAATGGCATAGACTTCTCTTTCATGACAAAAAAATGGTGGAAAAAAAGCTAGAAAGTTTAAGAGAATACGAAAGGAAAAGAATGCCTCCACGAGGAACTGATGTTTCTAGCTCTGAGCAGGACAATTCTTTCAAAATATCGACGCCAACAAAATCGTATGTTTCTTTGGAGCAAAAACCCTTACCAAATCTCTCTGCTATGAATAACTTTAATGATGTTACCGACAATAAGGAGAAAGAAGAAACGAACAACAATATATTAAAGTTCCAAGCGCAACGAGATCCATTACAAATACTACAGTCTGAGATCGAAATGCATACTAAGAAACTTGACACGATAATAGAGTTACTAAAAGACGATACCGATTCAAAGGAAAAAAGGAAAGTAGTGACTAATGACAACGCAGCGCCTGAACAAATGGTCAACAAAGGATGGCGGAAAAACGTGATGATGATCTACAAAAAATCAGGAAATATTATGAAAAAGTATAGGGAATATTTCTTATGGACAATTTGTATTTTAATATTGTTATATTGCAATATATATGTGTATTATAGGTTTTAAACCATATAAAGGTTAGTACATGAAGAAGTGTGTACAGTTATGGCAATAACTGTTCTCTTCCGTTTTAAAGAGAGTATTATCACGGTCTGAAAATTAAGCAAAAAAAGAAAAGCGTGCACTTAAAAATCGGTGGTGGACAGACCGCATATTCCATAAACATAGATGGTACAAGGGCATTATGAGTCCTTGAATAGAAAAAATGATGAAAGAATGAATGCAATTCCGTATTGCGACACTGGCGAAGTGTTCGAGGCTGACACGATAGCGAATGTATGGAAAAGAGAAGATAAGGAATGGTTAAAAAGGACTCAAAGTGATCGAAGTGGATACGAGTATCCACGACTAGGAGAATCACCATATATCAATATGACAGACGACTTCAGAGCGGCCGCCACCGCGGTGGAGCTCCAGCTTTTGTTCCCTTTAGTGAGGGTTAATTGCGCGCTTGGCGTAATCATGGTCATAGCTGTTTCCTGTGTGAAATTGTTATCCGCTCACAATTCCACACAACATAGGAGCCGGAAGCATAAAGTGTAAAGCCTGGGGTGCCTAATGAGTGAGGTAACTCACATTAATTGCGTTGCGCTCACTGCCCGCTTTCCAGTCGGGAAACCTGTCGTGCCAGCTGCATTAATGAATCGGCCAACGCGCGGGGAGAGGCGGTTTGCGTATTGGGCGCTCTTCCGCTTCCTCGCTCACTGACTCGCTGCGCTCGGTCGTTCGGCTGCGGCGAGCGGTATCAGCTCACTCAAAGGCGGTAATACGGTTATCCACAGAATCAGGGGATAACGCAGGAAAGAACATGTGAGCAAAAGGCCAGCAAAAGGCCAGGAACCGTAAAAAGGCCGCGTTGCTGGCGTTTTTCCATAGGCTCCGCCCCCCTGACGAGCATCACAAAAATCGACGCTCAAGTCAGAGGTGGCGAAACCCGACAGGACTATAAAGATACCAGGCGTTTCCCCCTGGAAGCTCCCTCGTGCGCTCTCCTGTTCCGACCCTGCCGCTTACCGGATACCTGTCCGCCTTTCTCCCTTCGGGAAGCGTGGCGCTTTCTCATAGCTCACGCTGTAGGTATCTCAGTTCGGTGTAGGTCGTTCGCTCCAAGCTGGGCTGTGTGCACGAACCCCCCGTTCAGCCCGACCGCTGCGCCTTATCCGGTAACTATCGTCTTGAGTCCAACCCGGTAAGACACGACTTATCGCCACTGGCAGCAGCCACTGGTAACAGGATTAGCAGAGCGAGGTATGTAGGCGGTGCTACAGAGTTCTTGAAGTGGTGGCCTAACTACGGCTACACTAGAAGGACAGTATTTGGTATCTGCGCTCTGCTGAAGCCAGTTACCTTCGGAAAAAGAGTTGGTAGCTCTTGATCCGGCAAACAAACCACCGCTGGTAGCGGTGGTTTTTTTGTTTGCAAGCAGCAGATTACGCGCAGAAAAAAAGGATCTCAAGAAGATCCTTTGATCTTTTCTACGGGGTCTGACGCTCAGTGGAACGAAAACTCACGTTAAGGGATTTTGGTCATGAGATTATCAAAAAGGATCTTCACCTAGATCCTTTTAAATTAAAAATGAAGTTTTAAATCAATCTAAAGTATATATGAGTAAACTTGGTCTGACAGTTACCAATGCTTAATCAGTGAGGCACCTATCTCAGCGATCTGTCTATTTCGTTCATCCATAGTTGCCTGACTCCCCGTCGTGTAGATAACTACGATACGGGAGGGCTTACCATCTGGCCCCAGTGCTGCAATGATACCGCGAGACCCACGCTCACCGGCTCCAGATTTATCAGCAATAAACCAGCCAGCCGGAAGGGCCGAGCGCAGAAGTGGTCCTGCAACTTTATCCGCCTCCATCCAGTCTATTAATTGTTGCCGGGAAGCTAGAGTAAGTAGTTCGCCAGTTAATAGTTTGCGCAACGTTGTTGCCATTGCTACAGGCATCGTGGTGTCACGCTCGTCGTTTGGTATGGCTTCATTCAGCTCCGGTTCCCAACGATCAAGGCGAGTTACATGATCCCCCATGTTGTGCAAAAAAGCGGTTAGCTCCTTCGGTCCTCCGATCGTTGTCAGAAGTAAGTTGGCCGCAGTGTTATCACTCATGGTTATGGCAGCACTGCATAATTCTCTTACTGTCATGCCATCCGTAAGATGCTTTTCTGTGACTGGTGAGTACTCAACCAAGTCATTCTGAGAATAGTGTATGCGGCGACCGAGTTGCTCTTGCCCGGCGTCAATACGGGATAATACCGCGCCACATAGCAGAACTTTAAAAGTGCTCATCATTGGAAAACGTTCTTCGGGGCGAAAACTCTCAAGGATCTTACCGCTGTTGAGATCCAGTTCGATGTAACCCACTCGTGCACCCAACTGATCTTCAGCATCTTTTACTTTCACCAGCGTTTCTGGGTGAGCAAAAACAGGAAGGCAAAATGCCGCAAAAAAGGGAATAAGGGCGACACGGAAATGTTGAATACTCATACTCTTCCTTTTTCAATATTATTGAAGCATTTATCAGGGTTATTGTCTCATGAGCGGATACATATTTGAATGTATTTAGAAAAATAAACAAATAGGGGTTCCGCGCACATTTCCCCGAAAAGTGCCACCTGGGTCCTTTTCATCACGTGCTATAAAAATAATTATAATTTAAATTTTTTAATATAAATATATAAATTAAAAATAGAAAGTAAAAAAAGAAATTAAAGAAAAAATAGTTTTTGTTTTCCGAAGATGTAAAAGACTCTAGGGGGATCGCCAACAAATACTACCTTTTATCTTGCTCTTCCTGCTCTCAGGTATTAATGCCGAATTGTTTCATCTTGTCTGTGTAGAAGACCACACACGAAAATCCTGTGATTTTACATTTTACTTATCGTTAATCGAATGTATATCTATTTAATCTGCTTTTCTTGTCTAATAAATATATATGTAAAGTACGCTTTTTGTTGAAATTTTTTAAACCTTTGTTTATTTTTTTTTCTTCATTCCGTAACTCTTCTACCTTCTTTATTTACTTTCTAAAATCCAAATACAAAACATAAAAATAAATAAACACAGAGTAAATTCCCAAATTATTCCATCATTAAAAGATACGAGGCGCGTGTAAGTTACAGGCAAGCGATCCGTCCTAAGAAACCATTATTATCATGACATTAACCTATAAAAATAGGCGTATCACGAGGCCCTTTCGTCTCGCGCGTTTCGGTGATGACGGTGAAAACCTCTGACACATGCAGCTCCCGGAGACGGTCACAGCTTGTCTGTAAGCGGATGCCGGGAGCAGACAAGCCCGTCAGGGCGCGTCAGCGGGTGTTGGCGGGTGTCGGGGCTGGCTTAACTATGCGGCATCAGAGCAGATTGTACTGAGAGT-3’  URA3 promoter-URA3 cassette-URA3 terminator-f1 ori-Left homologous arm-*KarI*Δ15 cassette-Right homology arm-ori-AmpR cassette-AmpR promoter-CEN/ARS |

Tab. 7 Plasmids used in this study.
