# Supplementary material for: Chromosome drives via CRISPR-Cas9 in yeast
Source: Nat Commun. 2020 Aug 28;11:4344. doi: 10.1038/s41467-020-18222-0 (PMC7455567; doi:10.1038/s41467-020-18222-0)
Supplement: Supplementary file 7 — Reporting Summary [file 41467_2020_18222_MOESM7_ESM.pdf]

nature research

Corresponding author(s): Yi Wu

Last updated by author(s): Aug 3, 2020

Reporting Summary

Nature Research wishes to improve the reproducibility of the work that we publish. This form provides structure for consistency and transparency in reporting. For further information on Nature Research policies, see our Editorial Policies and the Editorial Policy Checklist.

Statistics

For all statistical analyses, confirm that the following items are present in the figure legend, table legend, main text, or Methods section.

n/a	Confirmed	
☐	☒	The exact sample size (*n*) for each experimental group/condition, given as a discrete number and unit of measurement
☐	☒	A statement on whether measurements were taken from distinct samples or whether the same sample was measured repeatedly
☒	☐	The statistical test(s) used AND whether they are one- or two-sided<br>*Only common tests should be described solely by name; describe more complex techniques in the Methods section.*
☐	☒	A description of all covariates tested
☐	☒	A description of any assumptions or corrections, such as tests of normality and adjustment for multiple comparisons
☐	☒	A full description of the statistical parameters including central tendency (e.g. means) or other basic estimates (e.g. regression coefficient) AND variation (e.g. standard deviation) or associated estimates of uncertainty (e.g. confidence intervals)
☒	☐	For null hypothesis testing, the test statistic (e.g. *F*, *t*, *r*) with confidence intervals, effect sizes, degrees of freedom and *P* value noted<br>*Give P values as exact values whenever suitable.*
☒	☐	For Bayesian analysis, information on the choice of priors and Markov chain Monte Carlo settings
☒	☐	For hierarchical and complex designs, identification of the appropriate level for tests and full reporting of outcomes
☒	☐	Estimates of effect sizes (e.g. Cohen's *d*, Pearson's *r*), indicating how they were calculated

Our web collection on statistics for biologists contains articles on many of the points above.

Software and code

Policy information about availability of computer code

Data collection	No customised software was used.
Data analysis	No customised software was used.

For manuscripts utilizing custom algorithms or software that are central to the research but not yet described in published literature, software must be made available to editors and reviewers. We strongly encourage code deposition in a community repository (e.g. GitHub). See the Nature Research guidelines for submitting code & software for further information.

Data

Policy information about availability of data

All manuscripts must include a data availability statement. This statement should provide the following information, where applicable:
- Accession codes, unique identifiers, or web links for publicly available datasets
- A list of figures that have associated raw data
- A description of any restrictions on data availability

All sequencing data that support the findings of this study have been deposited into GeneBank (https://www.ncbi.nlm.nih.gov/genbank/) that are available from the Sequence Read Archive under Accession Code SRP269526 [https://www.ncbi.nlm.nih.gov/sra/?term=SRP269526].

Field-specific reporting

Life sciences study design

All studies must disclose on these points even when the disclosure is negative.

Sample size	No statistical methods were used to predetermine sample size. For PCRtag analysis of chromosome elimination efficiency, at least 96 samples was used for each experiment. In the experiment of chromosome drive with synthetic violacein pathway, 70 samples was chosen to test the efficiency. For high-throughput experiments, total 57 samples were chosen to verify the chromosome elimination and off-target event. For southern blot, gel electrophoresis and pulsed-field gel electrophoresis, at least 3 replicates were used for the experiment.
Data exclusions	No data were excluded from the analyses.
Replication	For the experiment of chromosome elimination efficiency of synX, 3 replicates of total 360 samples were chosen. For fluorescent protein experiment, at least three tetrads was observed that presented good consistency. For whole-genome sequencing experiments, due to its time and cost, 10 replicates were chosen for the analysis and one sample was chosen for the analysis of chromosome elimination, coupled with PCRtag and southern blot analysis, it can be a strong evidence for chromosome elimination. For southern blot, gel electrophoresis and pulsed-field gel electrophoresis, at least 3 replicates were used for the experiment.
Randomization	For the analysis of efficiency of chromosome elimination and off-target events, biological samples were chosen at random.
Blinding	Blinding is not relevant because no group allocation was involved in this study.

Reporting for specific materials, systems and methods

We require information from authors about some types of materials, experimental systems and methods used in many studies. Here, indicate whether each material, system or method listed is relevant to your study. If you are not sure if a list item applies to your research, read the appropriate section before selecting a response.

Materials & experimental systems

n/a	Involved in the study	
☒	☐	Antibodies
☐	☒	Eukaryotic cell lines
☒	☐	Palaeontology and archaeology
☒	☐	Animals and other organisms
☒	☐	Human research participants
☒	☐	Clinical data
☒	☐	Dual use research of concern

Methods

n/a	Involved in the study	
☒	☐	ChIP-seq
☒	☐	Flow cytometry
☒	☐	MRI-based neuroimaging

Eukaryotic cell lines

Policy information about cell lines

Cell line source(s)	No cell lines were used.
Authentication	No cell lines were authenticated.
Mycoplasma contamination	No cell lines were not tested for mycoplasma contamination.
Commonly misidentified lines (See ICLAC register)	No commonly misidentified cell lines were used.
